# A stabilization rule for metal carbido cluster bearing μ_3_-carbido single-atom-ligand encapsulated in carbon cage

**DOI:** 10.1038/s41467-023-44567-3

**Published:** 2024-01-02

**Authors:** Runnan Guan, Jing Huang, Jinpeng Xin, Muqing Chen, Pingwu Du, Qunxiang Li, Yuan-Zhi Tan, Shangfeng Yang, Su-Yuan Xie

**Affiliations:** 1 Key Laboratory of Precision and Intelligent Chemistry, Collaborative Innovation Center of Chemistry for Energy Materials (iChEM), Department of Materials Science and Engineering, University of Science and Technology of China, Hefei, 230026 China; 2 Hefei National Laboratory for Physical Sciences at Microscale, Department of Chemical Physics, Synergetic Innovation Center of Quantum Information & Quantum Physics, University of Science and Technology of China, Hefei, 230026 China; 3 School of Materials and Chemical Engineering, Anhui Jianzhu University, Hefei, 230601 China; 4 State Key Lab for Physical Chemistry of Solid Surfaces, Collaborative Innovation Center of Chemistry for Energy Materials (iChEM), Department of Chemistry, College of Chemistry and Chemical Engineering, Xiamen University, Xiamen, 361005 China

**Keywords:** Chemical bonding, Carbon nanotubes and fullerenes, Carbon nanotubes and fullerenes

## Abstract

Metal carbido complexes bearing single-carbon-atom ligand such as nitrogenase provide ideal models of adsorbed carbon atoms in heterogeneous catalysis. Trimetallic μ_3_-carbido clusterfullerenes found recently represent the simplest metal carbido complexes with the ligands being only carbon atoms, but only few are crystallographically characterized, and its formation prerequisite is unclear. Herein, we synthesize and isolate three vanadium-based μ_3_-CCFs featuring V = C double bonds and high valence state of V (+4), including VSc_2_C@*I*_*h*_(7)-C_80_, VSc_2_C@*D*_5*h*_(6)-C_80_ and VSc_2_C@*D*_3*h*_(5)-C_78_. Based on a systematic theoretical study of all reported μ_3_-carbido clusterfullerenes, we further propose a supplemental Octet Rule, i.e., an eight-electron configuration of the μ_3_-carbido ligand is needed for stabilization of metal carbido clusters within μ_3_-carbido clusterfullerenes. Distinct from the classic Effective Atomic Number rule based on valence electron count of metal proposed in the 1920s, this rule counts the valence electrons of the single-carbon-atom ligand, and offers a general rule governing the stabilities of μ_3_-carbido clusterfullerenes.

## Abstract (web summary)

Trimetallic carbido clusterfullerenes (CCFs) represent the simplest metal carbido complexes with the ligands being only carbon atoms, but the formation prerequisite is unclear. Herein, the authors report the syntheses of three novel vanadium(V)-based CCFs featuring high vanadium valence state and propose a supplemental Octet Rule

## Introduction

Organometallic complexes play a crucial role in catalysis, energy and medicine nowadays. Stabilities of organometallic complexes have been commonly determined by Effective Atomic Number (EAN) rule (i.e., 18-electron rule) proposed in the 1920s, that the effective atomic number of the central metal atom surrounded by ligands is numerically equal to the atomic number of the noble-gas element found in the same period as the metal^1^. EAN rule is based on valence electron count of the central metal atom instead of the non-metal ligand, and is applicable for a majority of organometallic complexes. In particular, metal carbido complexes bearing single-carbon-atom ligand such as the active site of nitrogenase (Fe_7_MoS_9_C) provide ideal models of adsorbed carbon atoms in heterogeneous catalysis, and have been attracting enormous interests during the past few decades^2–10^. Unlike the traditional multinuclear organometallic complexes, for metal carbido complexes the single-carbon-atom ligand becomes the center and bonds with 1 to 6 metals, thus the EAN rule is inapplicable due to the complicated coordination nature of the ligands especially the central single-carbon-atom ligand^2–10^. Hence, it is desirable to establish a new rule governing the stabilities of metal carbido complexes. For binuclear metal carbido complexes containing a carbido bridge such as L_n_M = C = ML_n_ and L_n_M ≡ C-M’L_n_, the central μ_2_-carbido ligand adopts an eight-electron configuration^4,5^. However, upon increasing the number of metals coordinated with the central single-carbon-atom ligand to three, the central μ_3_-carbido ligand does not always follow the eight-electron configuration^6–8^. Furthermore, in the metal carbido complexes bearing μ_5_- and μ_6_-carbido ligands, the central carbon atom is regarded as a hypervalency carbon due to the formation of more than four metal-carbon bonds^9,10^. Therefore, due to the diversity of the coordination numbers of the central single-carbon-atom ligand, it is difficult to establish a general rule for the conventional metal carbido complexes.

As the simplest metal carbido complexes with the ligands being only carbon atoms, trimetallic μ_3_-carbido clusterfullerenes (μ_3_-CCFs) featuring confinement of a single-carbon-atom ligand within carbon cage was found in 2014 (ref. ^11^). Due to electron transfer from the encapsulated metal carbido cluster to the outer carbon cage, μ_3_-CCFs exhibit intriguing electronic properties and promising applications in spintronics and high-density storage devices inaccessible by the conventional metal carbido complexes^11–19^. TiLu_2_C@*I*_*h*_(7)-C_80_ is the first μ_3_-CCF isolated in 2014, in which a Ti=C double bond was identified by single-crystal X-ray diffraction^11^. Later on, a few Ti-based μ_3_-CCFs were isolated, including TiM_2_C@*I*_*h*_(7)-C_80_ (M = Sc, Y, Nd, Gd, Tb, Dy, Er)^11–16^, TiM_2_C@*D*_5*h*_(6)-C_80_ (M = Sc, Dy)^12,13^, and TiSc_2_C@C_78_ (ref. ^13^), among which only TiSc_2_C@*I*_*h*_(7)-C_80_, TiDy_2_C@*I*_*h*_(7)-C_80_ and TiTb_2_C@*I*_*h*_(7)-C_80_ were crystallographically determined. More recently, another non-rare earth (non-RE) metal, the actinide metal uranium (U), was also reported to form μ_3_-CCF USc_2_C@*I*_*h*_(7)-C_80_, in which the U metal exhibits a formal valence state of +4 and bonds with the central μ_3_-carbido ligand via a U = C double bond as well^17,18^. Different to the well-known trimetallic nitride clusterfullerenes (NCFs) M_3_N@C_2n_ bearing primarily RE metals with +3 valence states (M^3+^) and M-N single bonds^20,21^, in μ_3_-CCFs the valence state of the non-RE metal changes to +4 as the result of formation of M = C (M = Ti, U) double bond, while the RE metals keep the +3 valence states and M-N single bonds^11–19^. Hence, μ_3_-CCFs offer a unique platform stabilizing μ_3_-carbido ligand which bonds with metals via multiple bonds. Although a number of μ_3_-CCFs have been isolated, only few were crystallographically characterized, and the reported non-RE metals within μ_3_-CCFs are quite limited to Ti and U. This limitation is because the formation prerequisite of μ_3_-CCF is unclear. Therefore, it is highly desired to explore new μ_3_-CCFs based on other non-RE metals and to establish a general rule elucidating stabilization of the μ_3_-carbido ligand within it.

Herein, we synthesized and isolated three vanadium(V)-based μ_3_-CCFs, including VSc_2_C@*I*_*h*_(7)-C_80_, VSc_2_C@*D*_5*h*_(6)-C_80_ and VSc_2_C@*D*_3*h*_(5)-C_78_, among them the latter two represent the first crystallographically determined non-*I*_*h*_-symmetry μ_3_-CCFs. The common feature of their molecular structures is the existence of V = C double bond along with Sc-C single bonds. Their electronic structures were investigated by density functional theory (DFT) calculations, unraveling high valence state of V (+4). Combining all reported sixteen μ_3_-CCFs, we further carried out a systematic DFT study on their stabilities, and proposed a supplemental Octet Rule based on valence electron count of the central ligand to account for stabilization of the metal carbido cluster within μ_3_-CCF. This rule is also applicable for NCF VSc_2_N@C_80_ and the conventional binuclear metal carbido complexes containing a carbido bridge such as L_n_M = C=ML_n_ and L_n_M ≡ C-M’L_n_, thus offers a general rule determining the stabilities of μ_3_-CCFs and guides the exploration of μ_3_-CCFs or even other metal carbido complexes.

## Results

### Syntheses and Isolation of VSc_2_C@C_80_ (I, II) and VSc_2_C@C_78_

V-based μ_3_-CCFs, including two isomers of VSc_2_C@C_80_ (labeled as I, II) and VSc_2_C@C_78_ were synthesized by Krätschmer-Huffman direct current (DC) arc discharge method^19^. Graphite rods packed with a mixture of Sc_2_O_3_, VC and graphite powder with a molar ratio of 0.5:1:15 were vaporized in the arcing chamber under a 200 mbar helium atmosphere. The obtained soot was then extracted with carbon disulfide (CS_2_), followed by four-step high-performance liquid chromatography (HPLC) isolations supplemented by laser desorption time-of-flight mass spectroscopic (LD-TOF MS) analysis. In the first step, fractions **A** and **B** both contain the same MS signal peak at M/Z = 1113 (Supplementary Fig. 1 and Table 1), which is assigned to VSc_2_C@C_80_. Since the retention times of fractions **A** and **B** are quite different, the two VSc_2_C@C_80_ molecules detected in these two fractions are isomers with different cage isomeric structures. The first isomer VSc_2_C@C_80_ (I) has been isolated from fraction **A** and identified as VSc_2_C@*I*_*h*_(7)-C_80_ very recently (Supplementary Fig. 2)^19^, therefore the second isomer isolated from fractions **B** after four-step HPLC separation is labeled as VSc_2_C@C_80_ (II) (Supplementary Fig. 3). Besides, another V-μ_3_-CCF with a MS signal peak at M/Z = 1089 is also isolated from fraction **C** (Supplementary Fig. 4), which is assigned to VSc_2_C@C_78_.

**Fig. 1:**
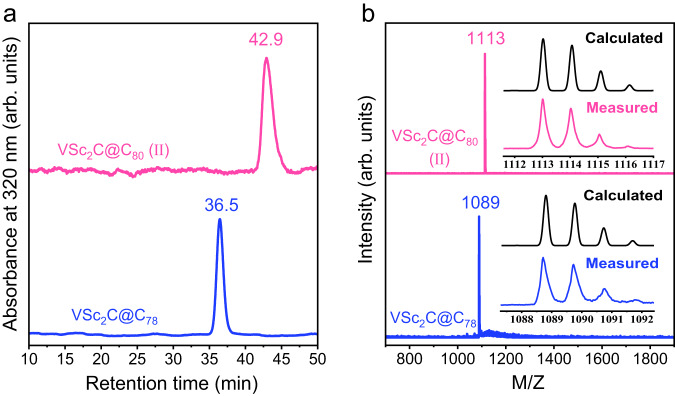
The purity examination of VSc_2_C@C_80_ (II) and VSc_2_C@C_78_. **a** HPLC chromatograms and **b** LD-TOF mass spectra of VSc_2_C@C_80_ (II) and VSc_2_C@C_78_. (column: Buckyprep, eluent: toluene, flow rate: 5 mL.min^−1^, injection volume: 5 mL, temperature: 40 °C).

The high purities of the isolated VSc_2_C@C_80_ (II) and VSc_2_C@C_78_ are verified by the single peaks observed by HPLC (Fig. 1a) and single mass peaks in their LD-TOF MS spectra (Fig. 1b). Furthermore, the isotopic distributions of VSc_2_C@C_80_ (II) and VSc_2_C@C_78_ agree well with the calculated ones, confirming their proposed chemical formulae. Interestingly, the analogous V-based μ_3_-CCF V_2_ScC@C_80_ and Sc-only μ_3_-CCF Sc_3_C@C_80_ are not detected, and this phenomenon is distinctly different from the case of the reported V-based NCFs for which both VSc_2_N@C_80_ and V_2_ScN@C_80_ were synthesized along with Sc_3_N@C_80_^22,23^. The difference between V-based μ_3_-CCFs and NCFs suggests the unique formation prerequisite of μ_3_-CCF as discussed in details below.

### X-ray crystallographic structures of VSc_2_C@*D*_5*h*_(6)-C_80_ and VSc_2_C@*D*_3*h*_(5)-C_78_

**Fig. 2:**
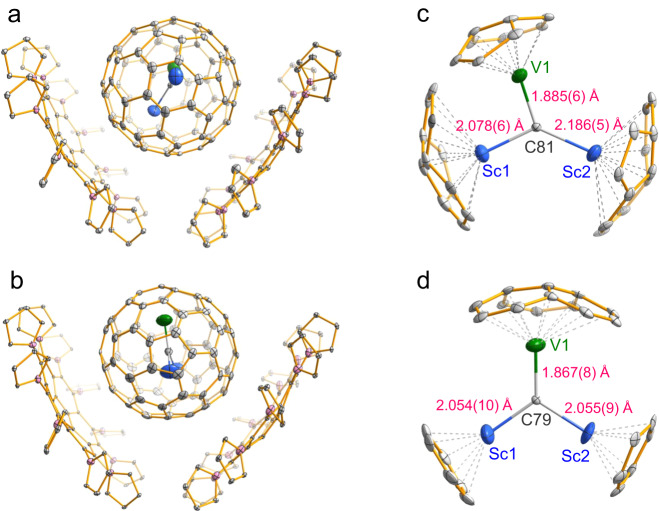
X-ray structures of VSc_2_C@*D*_5*h*_(6)-C_80_ and VSc_2_C@*D*_3*h*_(5)-C_78_. Drawings of the crystallographically determined structures of VSc_2_C@*D*_5*h*_(6)-C_80_·2(DPC) (**a**) and VSc_2_C@*D*_3*h*_(5)-C_78_·2(DPC) (**b**). The positions of the major cluster sites with respect to the nearest carbon atoms of cage within VSc_2_C@*D*_5*h*_(6)-C_80_ (**c**) and VSc_2_C@*D*_3*h*_(5)-C_78_ (**d**). Only one orientation of the fullerene cage together with the major site of VSc_2_C cluster is given for clarity. Solvent molecules, the minor cage and minor metal positions are omitted for clarity. Gray: C; Blue: Sc; Green: V; Pink: N.

To determine the molecular structures of VSc_2_C@C_80_ (II) and VSc_2_C@C_78_, we used decapyrrylcorannulene (DPC) as a host to co-crystallize them^24–28^. Black single co-crystals were both obtained and used for single-crystal X-ray diffraction study, accomplishing unambiguous determination of their molecular structures as VSc_2_C@*D*_5*h*_(6)-C_80_ and VSc_2_C@*D*_3*h*_(5)-C_78_ (see Supplementary Tables 2–4 for the detailed crystallographic data and discussion). Figure 2a, b exhibit the molecular structures of these two μ_3_-CCFs together with DPC molecules within VSc_2_C@*D*_5h_(6)-C_80_·2(DPC)·4(C_7_H_8_) and VSc_2_C_2_@*D*_3*h*_(5)-C_78_·2(DPC)·4(C_7_H_8_) co-crystals, revealing that the DPC molecules adopt V-shape configuration and embrace two fullerene molecules. Such a stoichiometric ratio of 1:2 is quite different from the 1:1 ratio in the co-crystals of endohedral fullerenes with the commonly used Ni^II^(OEP) (OEP = octaethylporphyrin) host, suggesting their difference in host-guest interactions^29–33^. To date, all crystallographically determined μ_3_-CCFs, including TiM_2_C@*I*_*h*_(7)-C_80_ (M = Sc^13^, Tb^14^, Dy^16^, Lu^11^), USc_2_C@*I*_*h*_(7)-C_80_ (ref. ^18^), and VSc_2_C@*I*_*h*_(7)-C_80_ (ref. ^19^), are based on *I*_*h*_(7)-C_80_ cage. Although non-*I*_*h*_-symmetry cages such as TiSc_2_C@*D*_5*h*_(6)-C_80_, TiDy_2_C@*D*_5*h*_(6)-C_80_ and TiSc_2_C@*D*_3*h*_(5)-C_78_ have been isolated, none of them were crystallographically characterized^12,13^. Therefore, VSc_2_C@*D*_5*h*_(6)-C_80_ and VSc_2_C@*D*_3*h*_(5)-C_78_ represent the first non-*I*_*h*_-symmetry μ_3_-CCFs with the molecular structures unambiguously determined by X-ray crystallography.

The fullerene cages of VSc_2_C@*D*_5*h*_(6)-C_80_ and VSc_2_C@*D*_3*h*_(5)-C_78_ are disordered in two orientations. Similar to the cases of VSc_2_C@*I*_*h*_(7)-C_80_ and other EMFs^19,34^, the encapsulated V/Sc atoms within VSc_2_C cluster exhibit obvious disorders due to the thermal vibration, whereas the central carbon atoms are fully ordered. Although the encapsulated VSc_2_C clusters within VSc_2_C@*D*_5*h*_(6)-C_80_ and VSc_2_C@*D*_3*h*_(5)-C_78_ are both disordered in four orientations, V and Sc atoms can be distinguished according to a comparison of the R1/wR2 values obtained from different conformations of the encapsulated VSc_2_C cluster combined with DFT calculations (see Supplementary Tables 5, 6 and Figs. 5–7 for details). Close-up views of the molecular structures of VSc_2_C@*D*_5*h*_(6)-C_80_ and VSc_2_C@*D*_3*h*_(5)-C_78_ with only major orientation of the fullerene cage and the major site of VSc_2_C cluster are shown in Fig. 2c, d and Supplementary Fig. 8. For VSc_2_C@*D*_5*h*_(6)-C_80_, the V atom lies at the pentagon-hexagon conjunction, while the two Sc atoms are beneath the pentagon-hexagon-hexagon conjunctions. Upon decreasing the cage size to *D*_3*h*_(5)-C_78_, V atom is beneath the hexagon-hexagon-hexagon conjunction and two Sc atoms both reside under the center of hexagon. Despite of the difference on the locations of metal atoms, the VSc_2_C clusters within VSc_2_C@*D*_5*h*_(6)-C_80_ and VSc_2_C@*D*_3*h*_(5)-C_78_ both keep the planar triangle geometry since the sum of metal-carbon-metal angles is close to 360°. This feature resembles those of VSc_2_C@*I*_*h*_(7)-C_80_ (ref. ^19^) and other crystallographically determined MSc_2_C@*I*_*h*_(7)-C_80_ (M = Ti^13^, U^18^) μ_3_-CCFs.

The crystallographic characterizations of VSc_2_C@*D*_5*h*_(6)-C_80_ and VSc_2_C@*D*_3*h*_(5)-C_78_ facilitate analyses of V-C and Sc-C bonding natures. As illustrated in Fig. 2c,d, the lengths of V-C bond within VSc_2_C@*D*_5*h*_(6)-C_80_ and VSc_2_C@*D*_3*h*_(5)-C_78_ are 1.885(6) Å and 1.867(8) Å, respectively, which are comparable to that within VSc_2_C@*I*_*h*_(7)-C_80_ (1.877(5) Å)^19^ and thus can be assigned to V = C double bond. Interestingly, this feature differs from those of VSc_2_N@*I*_*h*_(7)-C_80_ and VSc_2_N@*D*_5*h*_(6)-C_80_ NCFs in which V-N single bonds exist instead^22,23^. On the other hand, the lengths of Sc-C bonds in VSc_2_C@*D*_5*h*_(6)-C_80_ and VSc_2_C@*D*_3*h*_(5)-C_78_ are respectively 2.078(6)/2.186(5) Å and 2.054(10)/2.055(9) Å (Supplementary Table 7), which are very close to those observed in MSc_2_C@*I*_*h*_(7)-C_80_ (M = V^19^, Ti^13^, U^18^), indicating that the two Sc–C bonds are single bonds. Notably, the metal-to-carbon distances in VSc_2_C@*D*_5*h*_(6)-C_80_ and VSc_2_C@*I*_*h*_(7)-C_80_ are all slightly larger than that in VSc_2_C@*D*_3*h*_(5)-C_78_, suggesting stretching of VSc_2_C cluster along with the cage expansion from C_78_ to C_80_. The V-C and Sc-C bonding features observed in VSc_2_C@*D*_5*h*_(6)-C_80_ and VSc_2_C@*D*_3*h*_(5)-C_78_ as well as VSc_2_C@*I*_*h*_(7)-C_80_ highly resemble those in the reported μ_3_-CCFs TiM_2_C@*I*_*h*_(7)-C_80_ (M = Sc^13^, Tb^14^, Dy^16^, Lu^11^) and USc_2_C@*I*_*h*_(7)-C_80_ (ref. ^18^), indicating the considerable structural similarity among μ_3_-CCFs in terms of the existence of one double bond between the non-RE metal and the central carbon atom along with two RE metal-C single bonds. This stimulates us to propose a stabilization mechanism of μ_3_-CCF as discussed below.

### DFT calculations of electronic configurations of VSc_2_C@*D*_5*h*_(6)-C_80_ and VSc_2_C@*D*_3*h*_(5)-C_78_

**Fig. 3:**
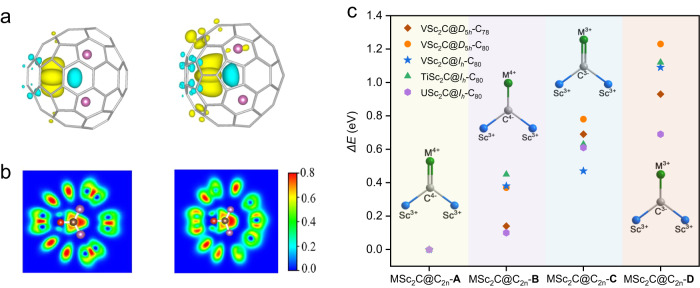
Electronic structures and relative total energies of VSc_2_C@*D*_5*h*_(6)-C_80_ and VSc_2_C@*D*_3*h*_(5)-C_78_. **a** Spin density distribution of VSc_2_C@*D*_5*h*_(6)-C_80_ (left) and VSc_2_C@*D*_3*h*_(5)-C_78_ (right) with an isovalue of 0.002. Yellow: V; Pink: Sc; Blue: C. **b** Schematic diagram for electronic localized functions (ELF) maps of VSc_2_C@*D*_5*h*_(6)-C_80_ (left) and VSc_2_C@*D*_3*h*_(5)-C_78_ (right) acting on the plane of the entrapped clusters. Red: V; Pink: Sc; Gray: C. **c** The relative total energies (ΔE, eV) of MSc_2_C@C_2n_ (M = V, Ti, U, 2n = 78, 80) μ_3_-CCFs with different electronic configurations of MSc_2_C cluster.

To investigate the electronic structures of VSc_2_C@*D*_5*h*_(6)-C_80_ and VSc_2_C@*D*_3*h*_(5)-C_78_ including the valence states of the encapsulated V atom and the interaction between V and C atoms, we carried out DFT calculations with the Vienna Ab-initio Simulation Package (VASP) at generalized gradient approximation (GGA) in the Perdew-Burke-Ernzerhof (PBE) levels^35^. According to DFT optimized molecular structures (Supplementary Fig. 9), the V-C bond lengths within VSc_2_C@*D*_5*h*_(6)-C_80_ and VSc_2_C@*D*_3*h*_(5)-C_78_ are 1.850 Å and 1.810 Å, respectively, while the Sc-C distances are 2.15/2.15 Å and 2.20/2.22 Å. These theoretical predictions agree well with the crystallographical results discussed above. To explore electronic structures of the VSc_2_C@*D*_5*h*_(6)-C_80_ and VSc_2_C@*D*_3*h*_(5)-C_78_, we calculated the spin-resolved molecular levels and plot the spatial distributions of several frontier molecular orbitals (Supplementary Fig. 10). It is clear that two molecules possess the spin-polarized ground states. Most frontier molecular orbitals are delocalized on the whole carbon cages, while there are also some localized molecular orbitals, mainly contributed by the inner VSc_2_C cluster. For example, for the spin-up electrons, the single occupied molecular orbitals (SOMO-1) of VSc_2_C@*D*_5*h*_(6)-C_80_ and SOMO-3 of VSc_2_C@*D*_3*h*_(5)-C_78_ mainly localized around the V atom, according to the percentage of the V occupations in the majority-orbital composition. These results imply that there is one unpaired electron for V atom in VSc_2_C@*D*_5*h*_(6)-C_80_ and VSc_2_C@*D*_3*h*_(5)-C_78_, leading to the doublet ground states. The total magnetic moments of VSc_2_C@*D*_5*h*_(6)-C_80_ and VSc_2_C@*D*_3*h*_(5)-C_78_ are predicted to be about 1.02 and 0.98 µ_B_, respectively. Figure 3a illustrates the spin-density spatial distributions of two molecules. Clearly, the spin-density mainly localized around the V atom, indicating that the V atom contributes mainly to the total magnetic moment. The atomic magnetic moment of V atom is about 1.15 and 1.05 µ_B_ in VSc_2_C@*D*_5*h*_(6)-C_80_ and VSc_2_C@*D*_3*h*_(5)-C_78_, respectively. Note that, in these two molecules the V atom antiferromagnetically couples with the neighboring C atom. The calculated partial density of states (DOS) for the V atom’s spin-split d orbitals of VSc_2_C@*D*_5*h*_(6)-C_80_ and VSc_2_C@*D*_3*h*_(5)-C_78_ display different distributions for the majority and minority electrons (Supplementary Fig. 11). The molecular magnetic moments with one unpaired electron are mainly contributed by the ${3d}_{{z}^{2}}$ orbitals of the V atom. At the same time, the ${3d}_{{xy}}$ and ${3d}_{{yz}}$ orbitals give the non-negligible contributions to total magnetic moments of VSc_2_C@*D*_5*h*_(6)-C_80_ and VSc_2_C@*D*_3*h*_(5)-C_78_, respectively. The electron configuration of the V atom is [Ar]3*d*^3^4*s*^2^, and one unpaired electron means the loss of four valence electrons from the V atom, resulting in a valence state of V^4+^ within both μ_3_-CCFs.

The V-C bonding type within VSc_2_C@*D*_5*h*_(6)-C_80_ and VSc_2_C@*D*_3*h*_(5)-C_78_ can be analyzed by the electronic localized function (ELF) maps, shown in Fig. 3b. Obviously, the interactions between V and C atoms are similar for two molecules. The visible electronic distributions are found on V atom, implying covalent interactions between V and C atoms. This is similar to the case of VSc_2_C@*I*_*h*_(7)-C_80_ (ref. ^19^). Note that the electronic distribution on the V atom in VSc_2_C@*D*_5*h*_(6)-C_80_ is slightly less than that in VSc_2_C@*D*_3*h*_(5)-C_78_. This observation is consistent with their difference on the predicted V-C distances (1.850 and 1.810 Å in VSc_2_C@*D*_5*h*_(6)-C_80_ and VSc_2_C@*D*_3*h*_(5)-C_78_, respectively). The different electronic distributions in these two molecules can be understood by the analysis of the Bader charges, since the electronic transfer from the V atom to the outer cage within VSc_2_C@*D*_5*h*_(6)-C_80_ (1.33 e) is larger than that within VSc_2_C@*D*_3*h*_(5)-C_78_ (1.30 e). Combining the analyses of the spin density, the frontier molecular orbitals, partial density of states, and ELF maps, a + 4 formal valence state of the encapsulated V and covalent interactions for V = C double bonds are revealed for both VSc_2_C@*D*_5*h*_(6)-C_80_ and VSc_2_C@*D*_3*h*_(5)-C_78_ μ_3_-CCFs. Noteworthy, The proposed V^4+^ configuration within VSc_2_C@*I*_*h*_(7)-C_80_ and VSc_2_C@*D*_5*h*_(6)-C_80_ μ_3_-C-CFs is obviously different to the V^3+^ state within the reported VSc_2_N@*I*_*h*_(7)-C_80_ and VSc_2_N@*D*_5*h*_(6)-C_80_ NCFs^22,23^. Hence, the valence state of V can be steered via simply altering the non-metal atom within the encapsulated metal cluster.

### Supplemental Octet Rule for μ_3_-carbido ligand in μ_3_-CCFs

Due to the multihapto nature of the carbon cage, EAN rule is inapplicable for μ_3_-CCFs. To understand the peculiar formation of the μ_3_-carbido ligand in μ_3_-CCFs, we carried out a systematic DFT study on the stabilities of VSc_2_C@*I*_*h*_(7)-C_80_, VSc_2_C@*D*_5*h*_(6)-C_80_ and VSc_2_C@*D*_3*h*_(5)-C_78_ with different electronic configurations based on M = C/M-C bonds with M^4+^/M^3+^ valence states, combined with those of the reported MSc_2_C@*I*_*h*_(7)-C_80_ (M=Ti, U) μ_3_-CCFs as the representative members based on other non-rare earth metals of Ti, U. As seen from Fig. 3c, clearly [V^4+^(Sc^3+^)_2_C^4-^]^6+^@[*I*_*h*_(7)-C_80_]^6-^, [V^4+^(Sc^3+^)_2_C^4-^]^6+^@[*D*_5*h*_(6)-C_80_]^6-^ and [V^4+^(Sc^3+^)_2_C^4-^]^6+^@[*D*_3*h*_(5)-C_78_]^6-^ based on M^4+^ = C^4-^ double bond are the most stable configurations with the lowest total energies (see also Supplementary Table 8). Altering the M^4+^ = C^4-^ double bond to M^4+^-C^4-^ single bond/M^3+^ = C^3-^ double bond/M^3+^-C^3-^ single bond results in increased total energies and consequently less stable configurations. Similar results are obtained for MSc_2_C@*I*_*h*_(7)-C_80_ (M = Ti, U) μ_3_-CCFs. Therefore, for MSc_2_C@C_2n_ (M = V, Ti, U; 2n = 80, 78) μ_3_-CCFs, M^4+^/Sc^3+^ cations and the central C^4−^ anion are needed, affording one M = C double bond along with two Sc-C single bonds. The entire MSc_2_C cluster then transfers six electrons to the outer fullerene cage, enabling stable μ_3_-CCF molecule. In this way, the central C^4-^ anion exhibits an eight-electron configuration. So far, there are sixteen μ_3_-CCFs in total have been reported, we further calculated the relative energies of the other eleven μ_3_-CCFs with different configurations. We find that, similar to the cases of MSc_2_C@C_2n_ (M = V, Ti, U, 2n = 78, 80), the configuration bearing a central carbon with an eight-electron configuration is the most stable structure for all μ_3_-CCFs (Supplementary Table 9). Based on these results, we propose a supplemental Octet Rule, that the central μ_3_-carbido ligand prefers to have eight electrons in the valence shell so as to be stabilized in μ_3_-CCF. In fact, the classic Octet Rule proposed by Lewis in 1916 has been commonly used for covalent compounds^36,37^, but never been used for metal carbido complexes. In this work, we manage to extend it to μ_3_-CCFs as a special type of metal carbido complexes, and succeed in interpreting the peculiar formation of the μ_3_-carbido ligand within μ_3_-CCFs. Noteworthy, this supplemental Octet Rule is also applicable for the conventional binuclear metal carbido complexes containing a carbido bridge such as L_n_M = C = ML_n_ and L_n_M ≡ C-M’L_n_, but it is not always valid for μ_3_-carbido ligand within the conventional trinuclear metal carbido complexes^4–8^. Furtheromore, in the metal carbido complexes bearing μ_5_- and μ_6_-carbido ligands, this Rule is not applicable any more because the central carbon atom is coordinated with more than four metals and thus is a hypervalency carbon^9,10^. For the well-known metal carbido complex Fe_7_MoS_9_C as the active site of nitrogenase, although a central C^4-^ anion also exists, it violates the supplemental Octet Rule because the central C^4-^ anion bonds with six iron atoms to form a Fe_6_C core^2,38^.

According to this supplemental Octet Rule, the necessity of involving a four-valency non-RE metal for the formation of MSc_2_C@C_2n_ μ_3_-CCFs can be easily understood, since a M^4+^ cation is demanded to accomplish M = C double bond whereas +4 valence state is generally not preferable for the RE metals^39^. This also accounts for the absence of Sc-only μ_3_-CCF Sc_3_C@C_80_ under our synthesis condition, which is theoretically predicted very recently as an unstable free radical with one unpaired electron on the cage derived from the formal five-electron transfer^40^, since Sc^4+^ cation is hardly accessible. Interestingly, once the one deficient electron in the outer cage of unstable Sc_3_C@C_80_ is compensated by encapsulating one hydrogen atom, Sc_3_CH@C_80_ with an electronic configuration of [(Sc^3+^)_3_C^4-^H^+^]^6+^@[C_80_]^6-^ forms and the supplemental Octet Rule is also satisfied for the central C^4-^ anion^41,42^. Likewise, V_2_ScC@C_80_ based on two V^4+^ cations would violate the Octet Rule and thus seems also impossible. Therefore, this supplemental Octet Rule may be used as a simple guide for design of μ_3_-CCFs, offering opportunity to encapsulate other non-rare earth metals into fullerene cage.

It is intriguing to examine the applicability of this supplemental Octet Rule in other types of clusterfullerenes. VSc_2_N@*I*_*h*_(7)-C_80_ NCF as an analogous V-containing trimetallic clusterfullerene is considered. Interestingly, upon changing the central non-metal atom from carbon to nitrogen, [V^3+^(Sc^3+^)_2_N^3-^]^6+^@[*I*_*h*_(7)-C_80_]^6-^ based on V^3+^-N^3-^ single bond becomes the most stable configuration (see Supplementary Table 10), as experimentally confirmed^22^. Since N atom has five valence electrons, upon formation of VSc_2_N@*I*_*h*_(7)-C_80_ NCF, V^3+^/Sc^3+^ cations along with three V-N/Sc-N single bonds exist, rendering an eight-electron configuration for the central N^3-^ anion as well. Therefore, this supplemental Octet Rule is also applicable for NCF.

### Electronic properties of VSc_2_C@*D*_5*h*_(6)-C_80_ and VSc_2_C@*D*_3*h*_(5)-C_78_

**Fig. 4:**
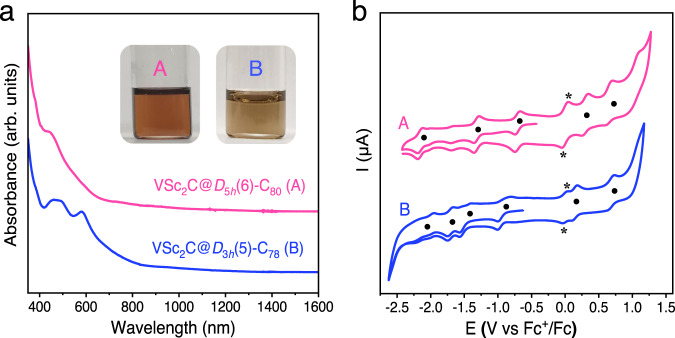
Electronic properties of VSc_2_C@*D*_5*h*_(6)-C_80_ and VSc_2_C@*D*_3*h*_(5)-C_78_. **a** UV-vis−NIR spectra of VSc_2_C@*D*_5*h*_(6)-C_80_ and VSc_2_C@*D*_3*h*_(5)-C_78_ dissolved in toluene. Insets: Photographs of their corresponding solutions in toluene. **b** Cyclic voltammograms of VSc_2_C@*D*_5*h*_(6)-C_80_ and VSc_2_C@*D*_3*h*_(5)-C_78_ in *o*-DCB solution with ferrocene (Fc) as the internal standard under different scan regions. Scan rate: 100 mV/s, TBAPF_6_ as supporting electrolyte. The half-wave potentials (E_1/2_) of each redox step are marked with a solid dot to aid comparison. The asterisk labels the oxidation and reduction peaks of ferrocene.

In order to probe the effect of the central nonmetal atom (C/N) on the electronic properties of μ_3_-CCF and NCF, we carried out UV-vis-NIR spectroscopic and electrochemical characterizations. Figure 4a compares the UV-vis−NIR absorption spectra of VSc_2_C@*D*_5*h*_(6)-C_80_ and VSc_2_C@*D*_3*h*_(5)-C_78_ dissolved in toluene (see Supplementary Fig. 12 and Table 11 for their characteristic absorption data along with analogous μ_3_-CCFs and NCFs). VSc_2_C@*D*_5*h*_(6)-C_80_ exhibits a broad absorption peak at 447 nm and a minor shoulder peak at 383 nm, and the overall spectral feature looks similar to that of VSc_2_N@*D*_5*h*_(6)-C_80_ but quite different from that of VSc_2_C@*I*_*h*_(7)-C_80_^22,23^. This is understandable since the outer fullerene cages of VSc_2_C@*D*_5*h*_(6)-C_80_ and VSc_2_N@*D*_5*h*_(6)-C_80_ are same while π − π* transitions of the fullerene cage predominantly determines the electronic absorptions of EMFs^34^. For VSc_2_C@*D*_3*h*_(5)-C_78_, two intense absorption peaks at 462 and 581 nm are observed, resembling Sc_3_N@*D*_3*h*_(5)-C_78_ despite of some shifts of the absorption peaks due to the discrepancy on the encapsulated cluster. According to the absorption spectral onsets of 1490 and 1430 nm for VSc_2_C@*D*_5*h*_(6)-C_80_ and VSc_2_C@*D*_3*h*_(5)-C_78_, their optical bandgaps (Δ*E*_gap, optical_) are determined to be 0.83 and 0.87 eV, respectively, which are comparable to that of VSc_2_C@*I*_*h*_(7)-C_80_ (0.88 eV)^19^.

Although few *D*_5*h*_(6)-C_80_- and *D*_3*h*_(5)-C_78_-based μ_3_-CCFs like TiSc_2_C@*D*_5*h*_(6)-C_80_, TiDy_2_C@*D*_5*h*_(6)-C_80_ and TiSc_2_C@*D*_3*h*_(5)-C_78_ were isolated before, their electrochemical properties have never been investigated yet. Figure 4b presents cyclic voltammograms of VSc_2_C@*D*_5*h*_(6)-C_80_ and VSc_2_C@*D*_3*h*_(5)-C_78_ measured in *o*-dichlorobenzene (*o*-DCB) with tetrabutylammonium hexafluorophosphate (TBAPF_6_) as supporting electrolyte (see Supplementary Fig. 13 for cyclic voltammograms in different scanning regions), and their characteristic redox potentials along with the analogous NCFs are summarized in Supplementary Table 12. For VSc_2_C@*D*_3*h*_(5)-C_78_, two reversible oxidation steps with half-wave potentials (E_1/2_) at 0.14 and 0.69 V in the anodic region are observed, and the first oxidation potential is more negative than that measured for Sc_3_N@*D*_3*h*_(5)-C_78_ while the second one is close to that of Sc_3_N@*D*_3*h*_(5)-C_78_ (ref. ^40^). In the cathodic region, VSc_2_C@*D*_3*h*_(5)-C_78_ shows quite different reductive behavior compared to Sc_3_N@*D*_3*h*_(5)-C_78_ in terms of number of the reduction steps: VSc_2_C@*D*_3*h*_(5)-C_78_ exhibits two irreversible reduction steps and two reversible reduction steps, while there are only two irreversible reduction steps for Sc_3_N@*D*_3*h*_(5)-C_78_ (ref. ^43^). In particular, the first reduction potential (^red^*E*_1_) of VSc_2_C@*D*_3*h*_(5)-C_78_ is positively shifted by 650 mV relative to that of Sc_3_N@*D*_3*h*_(5)-C_78_. The more positive ^red^*E*_1_ and more negative first oxidation potential (^ox^*E*_1_) of VSc_2_C@*D*_3*h*_(5)-C_78_ result in a much smaller electrochemical gap (1.05 eV) than that of Sc_3_N@*D*_3*h*_(5)-C_78_ (1.77 eV). Similar phenomenon is observed for VSc_2_C@*D*_5*h*_(6)-C_80_, which exhibits two reversible oxidation steps with E_1/2_ at 0.30 and 0.66 V and three reversible reduction steps with E_1/2_ at −0.70, −1.31, and −2.16 V. The ^ox^*E*_1_ and ^ox^*E*_2_ values are both more negative than those of VSc_2_C@*I*_*h*_(7)-C_80_ and VSc_2_N@*D*_5*h*_(6)-C_80_. In addition, the reductive behavior of VSc_2_C@*D*_5*h*_(6)-C_80_ is more different with VSc_2_N@*D*_5*h*_(6)-C_80_ which shows four reversible reduction steps instead, and the electrochemical gap of VSc_2_C@*D*_5*h*_(6)-C_80_ (1.00 eV) is smaller than that of VSc_2_N@*D*_5*h*_(6)-C_80_ (1.20 eV)^23^. Therefore, the encapsulated cluster especially the central nonmetal atom affects sensitively the electronic properties of the trimetallic clusterfullerene.

## Discussion

In summary, three V-based μ_3_-CCFs, namely VSc_2_C@*I*_*h*_(7)-C_80_, VSc_2_C@*D*_5*h*_(6)-C_80_ and VSc_2_C@*D*_3*h*_(5)-C_78_, are successfully synthesized and isolated, among them the latter two represent the first crystallographically determined non-*I*_*h*_-symmetry μ_3_-CCFs. Their molecular structures are determined unambiguously by single-crystal X-ray diffraction, revealing the existence of V = C double bonds and high valence state of V (+4). This differs from those of VSc_2_N@*I*_*h*_(7)-C_80_ and VSc_2_N@*D*_5*h*_(6)-C_80_ NCFs in which V-N single bonds and V^3+^ valence state exist. The encapsulated cluster especially the central nonmetal atom affects sensitively the electronic properties of μ_3_-CCF and NCF trimetallic clusterfullerenes. On the basis of a systematic DFT study on the stabilities of all reported sixteen μ_3_-CCFs, a supplemental Octet Rule is proposed, that the central μ_3_-carbido ligand prefers to have eight electrons in the valence shell so as to be stabilized in μ_3_-CCF. The applicability of this supplemental Octet Rule in other types of clusterfullerenes is exemplified by VSc_2_N@*I*_*h*_(7)-C_80_ NCF. By applying the classic Octet Rule to μ_3_-CCFs as the simplest metal carbido complexes, we establish a rule beyond the EAN rule commonly used during the past century, offering new insight into the stability criteria of multinuclear clusterfullerenes containing single-atom-ligand.

## Methods

### Synthesis and isolation of VSc_2_C@*I*_*h*_(7)-C_80_, VSc_2_C@*D*_5*h*_(6)-C_80_ and VSc_2_C@*D*_3*h*_(5)-C_78_

VSc_2_C@*I*_*h*_(7)-C_80_, VSc_2_C@*D*_5*h*_(6)-C_80_ and VSc_2_C@*D*_3*h*_(5)-C_78_ were synthesized in a Krätschmer-Huffman generator by vaporizing composite graphite rods containing a mixture of Sc_2_O_3_, VC and graphite powder (the molar ratio of Sc:V:C = 1:1:15) as the raw material with the addition of 200 mbar He. The produced soot was collected and Soxhlet-extracted by CS_2_ for 24 h. The resulting brown-yellow solution was distilled to remove CS_2_, and then immediately redissolved in toluene and subsequently passed through a 0.2 μm Telflon filter (Sartorius AG, Germany) for HPLC separation. I VSc_2_C@*I*_*h*_(7)-C_80_, VSc_2_C@*D*_5*h*_(6)-C_80_ and VSc_2_C@*D*_3*h*_(5)-C_78_ were isolated by three/four-step HPLC (LC-9104, Japan Analytical Industry) as described in details in Supplementary Figs. 1–4. The relative abundance of the products is shown in the Supplementary Table 1. The purity of the isolated VSc_2_C@*D*_5*h*_(6)-C_80_ and VSc_2_C@*D*_3*h*_(5)-C_78_ were checked by HPLC and LD-TOF MS (Biflex III, Bruker Daltonics Inc., Germany).

### Spectroscopic and electrochemical study

UV-vis−NIR spectra of VSc_2_C@*D*_5*h*_(6)-C_80_ and VSc_2_C@*D*_3*h*_(5)-C_78_ dissolved in toluene were recorded on a UV−vis−NIR 3600 spectrometer (Shimadzu, Japan) using a quartz cell of 1 mm layer thickness and 1 nm resolution. Electrochemical study of VSc_2_C@*D*_5*h*_(6)-C_80_ and VSc_2_C@*D*_3*h*_(5)-C_78_ were performed in *o*-dichlorobenzene (*o*-DCB, anhydrous, 99%, Aldrich). The supporting electrolyte was tetrabutylamonium hexafluorophosphate (TBAPF_6_, puriss. electrochemical grade, Fluka) which was dried under pressure at 340 K for 24 h and stored in glovebox prior to use. Cyclic voltammogram experiments were performed with a CHI 660D potentiostat (CHI Instrument, USA) at room temperature. A standard three-electrode arrangement of a platinum (Pt) disc as working electrode, a platinum wire as counter electrode, and a silver wire as an auxiliary electrode was used. In a comparison experiment, ferrocene (Fc) was added as the internal standard and all potentials are referred to Fc/Fc^+^ couple.

### X-ray crystallographic study

Crystal growths of VSc_2_C@*D*_5*h*_(6)-C_80_ and VSc_2_C@*D*_3*h*_(5)-C_78_ were accomplished by slow evaporation from mixed solutions of purified sample and DPC in toluene, and small black crystals suitable for X-ray crystallographic study were obtained after two weeks. The crystallographic characterization was performed in beamline station BL17B at Shanghai Synchrotron Radiation Facility. The structure was refined using all data (based on F^2^) by SHELXL 2015 (ref. ^44^) within OLEX2 (ref. ^45^). A summary of the crystallographic data is listed in Supplementary Table 2. The ORTEP-style illustration with probability ellipsoids and notes on CheckCif file B-level alerts are shown in Supplementary Fig. 14 and Supplementary Table 13.

### Computations

In our calculations, the geometrical structures and electronic properties were explored by performing spin-polarized density functional theory (DFT) methods implemented in the Vienna Ab-initio Simulation Package (VASP). The generalized gradient approximation (GGA) in the Perdew-Burke-Ernzerhof (PBE) form was adopted to describe the exchange and correlation energy^35^. The energy cutoff of 400 eV was selected for the plane wave expansion and an automatic k-point mesh (1 × 1 × 1) was generated with a Gamma-centered grid.

### Supplementary information

Supplementary Information

Peer Review File

## Data Availability

Crystallographic data of the structures reported in this Article have been deposited in the Cambridge Crystallographic Data Center (CCDC), under deposition numbers 2209496 (VSc_2_C@*D*_3*h*_(5)-C_78_·2(DPC)·3(C_7_H_8_)), 2038584 (VSc_2_C@*D*_5*h*_(6)-C_80_·2(DPC)·4(C_7_H_8_)) and 2038583 (VSc_2_C@*I*_*h*_(7)-C_80_·2(DPC)·3(C_7_H_8_)). Copies of the data can be obtained free of charge via https://www.ccdc.cam.ac.uk/structures/. All other data that support the findings of this study are available from the Supplementary Information and/or from the corresponding author upon request.
