# Supplementary material for: A stabilization rule for metal carbido cluster bearing μ3-carbido single-atom-ligand encapsulated in carbon cage
Source: Nat Commun. 2024 Jan 2;15:150. doi: 10.1038/s41467-023-44567-3 (PMC10761991; doi:10.1038/s41467-023-44567-3)
Supplement: Supplementary file 1 — Supplementary Information [file 41467_2023_44567_MOESM1_ESM.pdf]

SUPPLEMENTARY INFORMATION

A stabilization rule for metal carbido cluster bearing μ_3-carbido single-atom-ligand encapsulated in carbon cage

Runnan Guan,[a,†] *Jing Huang,*[b,d,†] *Jinpeng Xin,*[a] *Muqing Chen,*[a] *Pingwu Du,*[a] *Qunxiang Li*[b,*], *Yuan-Zhi Tan*[c,*], *Shangfeng Yang*[a,*] *and Su-Yuan Xie*[c]

[a] Key Laboratory of Precision and Intelligent Chemistry, Collaborative Innovation Center of Chemistry for Energy Materials (iChEM), Department of Materials Science and Engineering, University of Science and Technology of China, Hefei 230026, China

[b] Hefei National Laboratory for Physical Sciences at Microscale, Department of Chemical Physics, Synergetic Innovation Center of Quantum Information & Quantum Physics, University of Science and Technology of China, Hefei 230026, China

[c] State Key Lab for Physical Chemistry of Solid Surfaces, Collaborative Innovation Center of Chemistry for Energy Materials (iChEM), Department of Chemistry, College of Chemistry and Chemical Engineering, Xiamen University, Xiamen 361005, China

[d] School of Materials and Chemical Engineering, Anhui Jianzhu University, Hefei 230601, China

*Correspondence and requests for materials should be addressed to Q. L. (liqun@ustc.edu.cn), Y. T. (Email: yuanzhi_tan@xmu.edu.cn) and S. Y. (Email: sfyang@ustc.edu.cn).

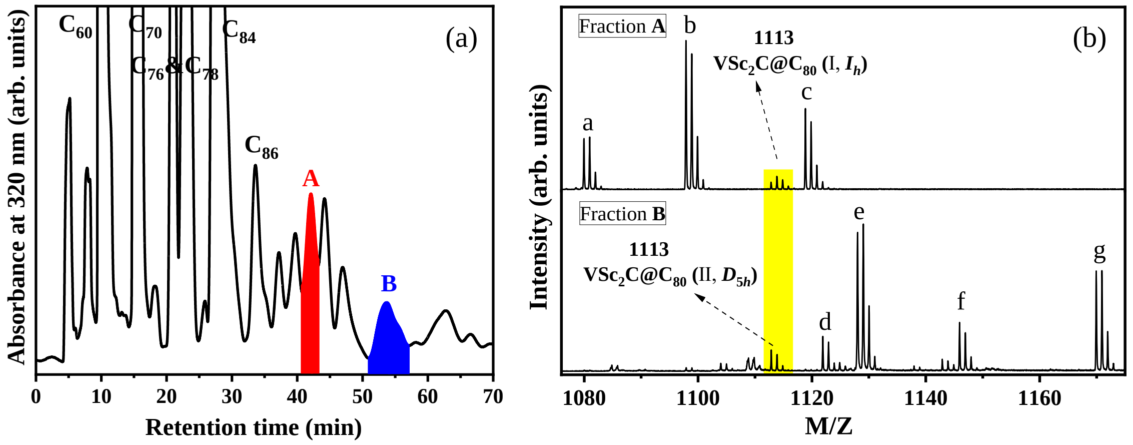

Supplementary Figure 1. (a) HPLC chromatogram of fullerene extract mixture. (b) LD-TOF mass spectra of fraction **A** and fraction **B** (HPLC conditions: column: 20 × 250 mm Buckyprep column, eluent: toluene, flow rate: 15 mL/min^{-1}, injection volume: 15 mL, temperature : 40 °C), Peaks a-c are assigned as C_{90}, Sc_2C_{84} and $Sc_3C_2@C_{80}$ in fraction **A**, peaks d-g are assigned as Sc_2C_{86}, C_{94}, Sc_2C_{88} and Sc_2C_{90} in fraction **B**.

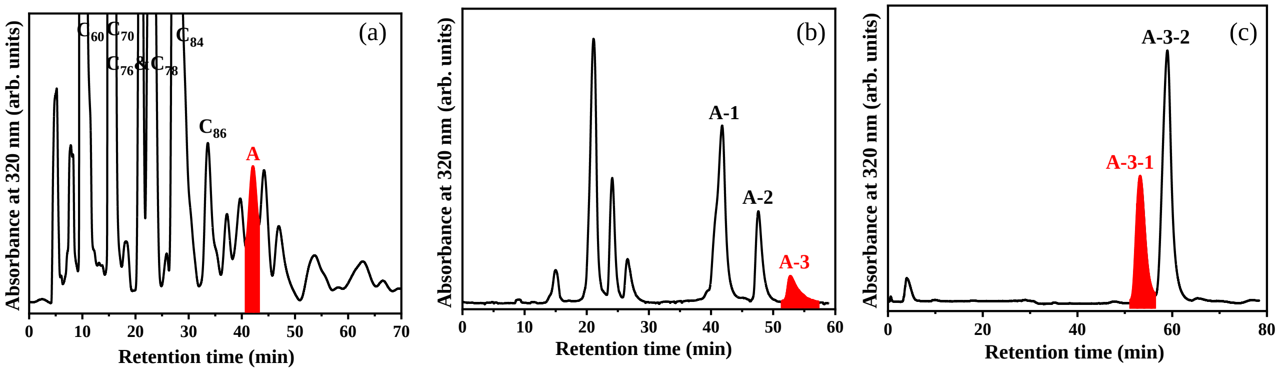

Supplementary Figure 2. Isolation of $VSc_2C@I_h(7)\text{-}C_{80}$ as $VSc_2C@C_{80}$ (I) (ref 1). (a) HPLC chromatogram of fullerene extract mixture by a 20 × 250 mm Buckyprep column, a flow rate of 15 mL/min, injection volume of 15 mL and toluene as the eluent at 40 °C. (b) HPLC chromatogram of fraction **A** obtained by a 20 × 250 mm Buckyprep-M column, a flow rate of 15 mL/min, injection volume of 15 mL and toluene as the eluent at 40 °C. (c) HPLC chromatogram of fraction **A-3** obtained by a 10 × 250 mm 5PBB column, a flow rate of 5 mL/min, injection volume of 5 mL and toluene as the eluent at 40 °C.

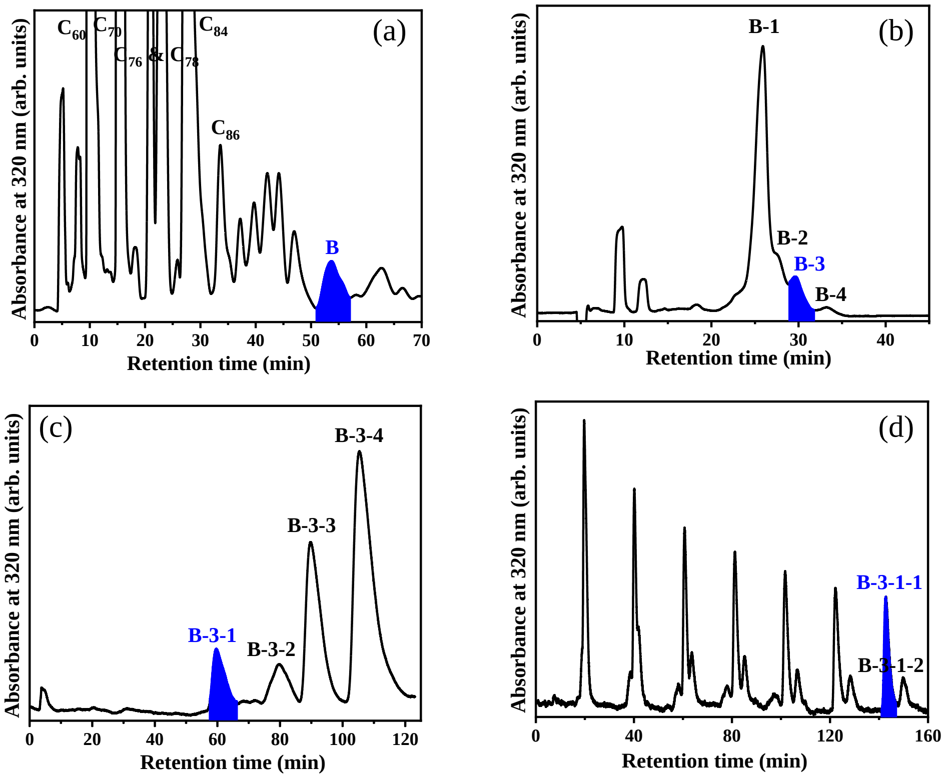

Supplementary Figure 3. Isolation of $VSc_2C@D_{5h}(6)$-C_{80} as $VSc_2C@C_{80}$ (II). (a) HPLC chromatogram of fullerene extract mixture by a 20 × 250 mm Buckyprep column, a flow rate of 15 mL/min, injection volume of 15 mL and toluene as the eluent at 40 ^{o}C. (b) HPLC chromatogram of fraction **B** obtained by a 20 × 250 mm Buckyprep-M column, a flow rate of 15 mL/min, injection volume of 15 mL and toluene as the eluent at 40 ^{o}C. (c) HPLC chromatogram of fraction **B-3** obtained by a 10 × 250 mm 5PBB column, a flow rate of 5 mL/min, injection volume of 5 mL and toluene as the eluent at 40 ^{o}C. (d) HPLC chromatogram of fraction **B-3-1** obtained by a 10 × 250 mm Buckyprep-M column, a flow rate of 5 mL/min, injection volume of 5 mL and toluene as the eluent at 40 ^{o}C.

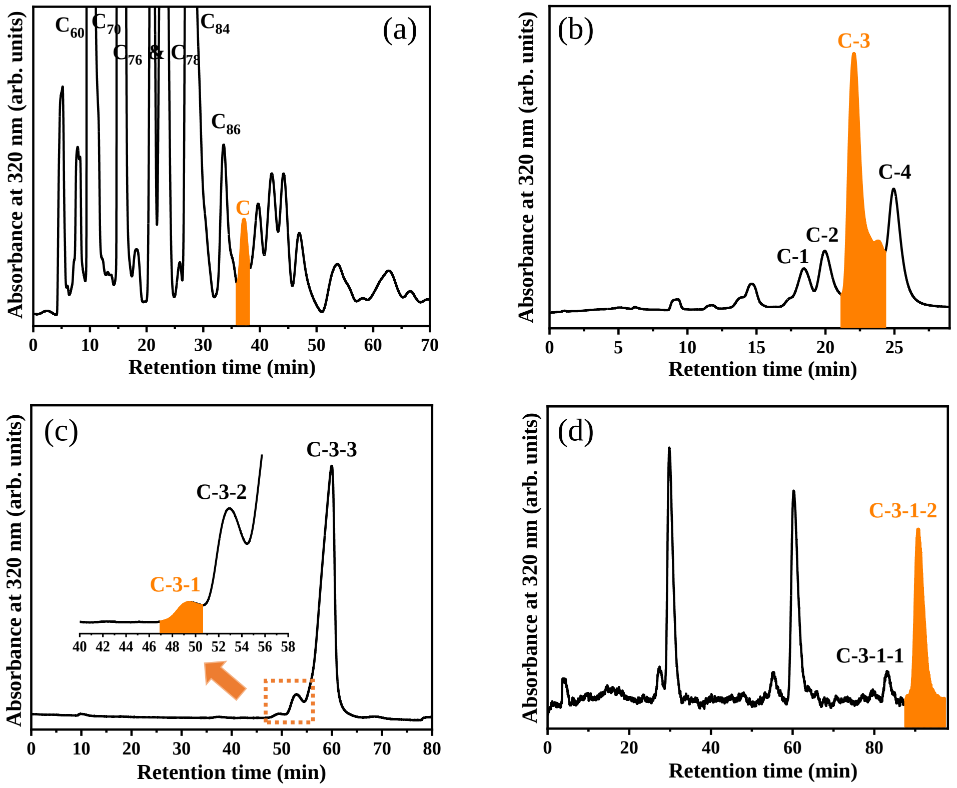

Supplementary Figure 4. Isolation of $VSc_2C@D_{3h}(5)$-C_{78}. (a) HPLC chromatogram of fullerene extract mixture obtained by a 20 × 250 mm Buckyprep column, a flow rate of 15 mL/min, injection volume of 15 mL and toluene as the eluent at 40 °C. (b) HPLC chromatogram of fraction **C** obtained by a 20 × 250 mm Buckyprep-M column, a flow rate of 15 mL/min, injection volume of 15 mL and toluene as the eluent at 40 °C. (c) HPLC chromatogram of fraction **C-3** obtained by a 10 × 250 mm 5PBB column, a flow rate of 5 mL/min, injection volume of 5 mL and toluene as the eluent at 40 °C. (d) HPLC chromatogram of fraction **C-3-1** obtained by a 10 × 250 mm Buckyprep column, a flow rate of 5 mL/min, injection volume of 5 mL and toluene as the eluent at 40 °C.

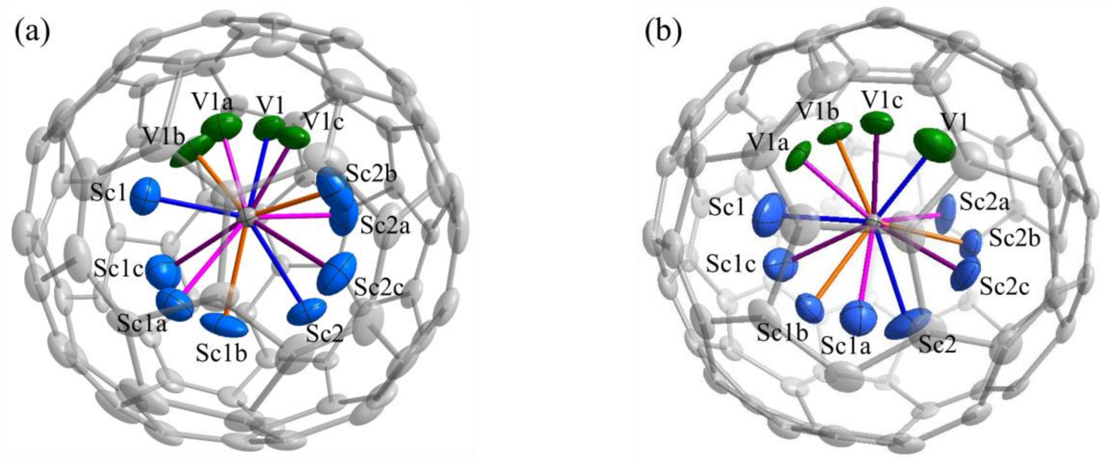

Supplementary Figure 5. Positions of the disordered Sc/V sites in $VSc_2C@D_{5h}(6)-C_{80}$ (a) and $VSc_2C@D_{3h}(5)-C_{78}$ (b) relative to the major cage orientation. The encapsulated VSc_2C clusters are both disordered in four orientations for $VSc_2C@D_{5h}(6)-C_{80}$ and $VSc_2C@D_{3h}(5)-C_{78}$. Interestingly, the VSc_2C clusters within $VSc_2C@D_{5h}(6)-C_{80}$ and $VSc_2C@D_{3h}(5)-C_{78}$ show roughly free rotation on the mirror plane of the two nearby DPC molecules, which is quite similar to the case of $VSc_2C@I_h(7)-C_{80}$ (ref. 1).

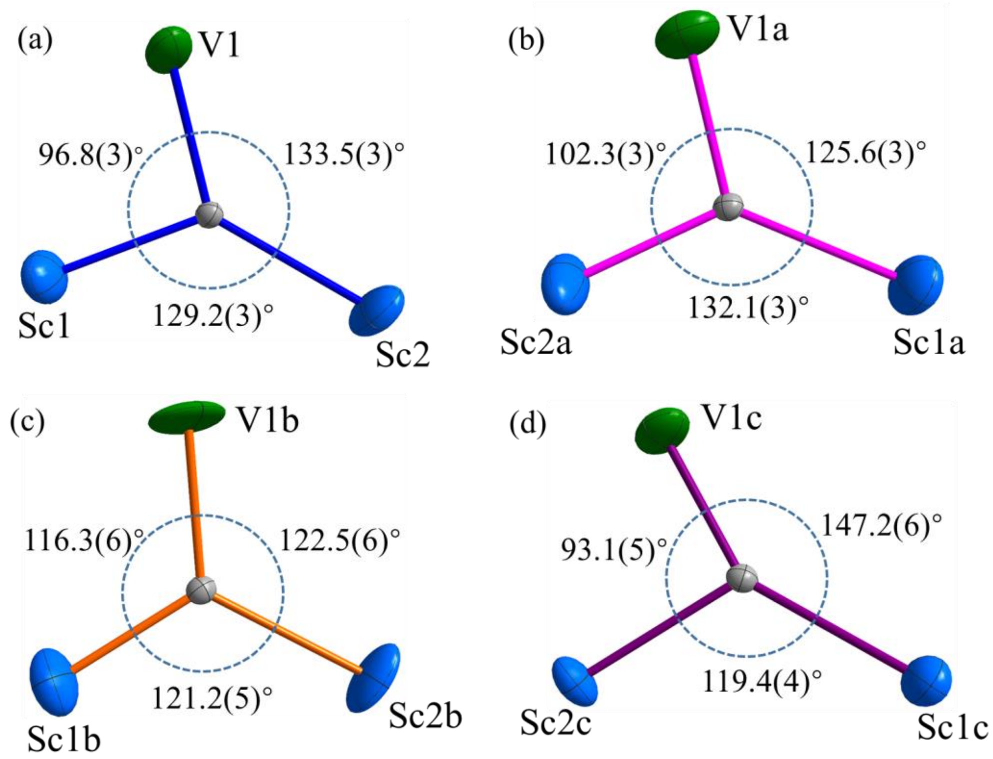

Supplementary Figure 6. Orientations of VSc_2C unit in $VSc_2C@D_{5h}(6)$-C_{80} including the major (a) and minor (b, c, d) orientations.

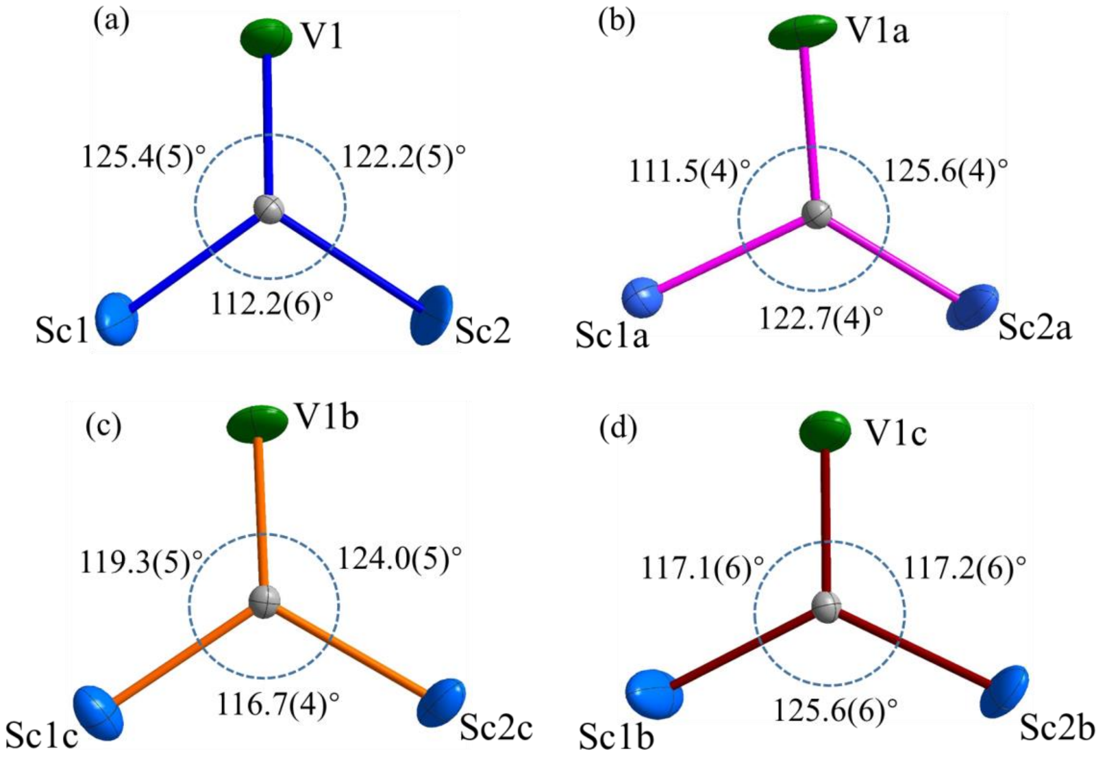

Supplementary Figure 7. Orientations of VSc_2C unit in $VSc_2C@D_{3h}(5)\text{-}C_{78}$ including the major (a) and minor (b, c, d) orientations.

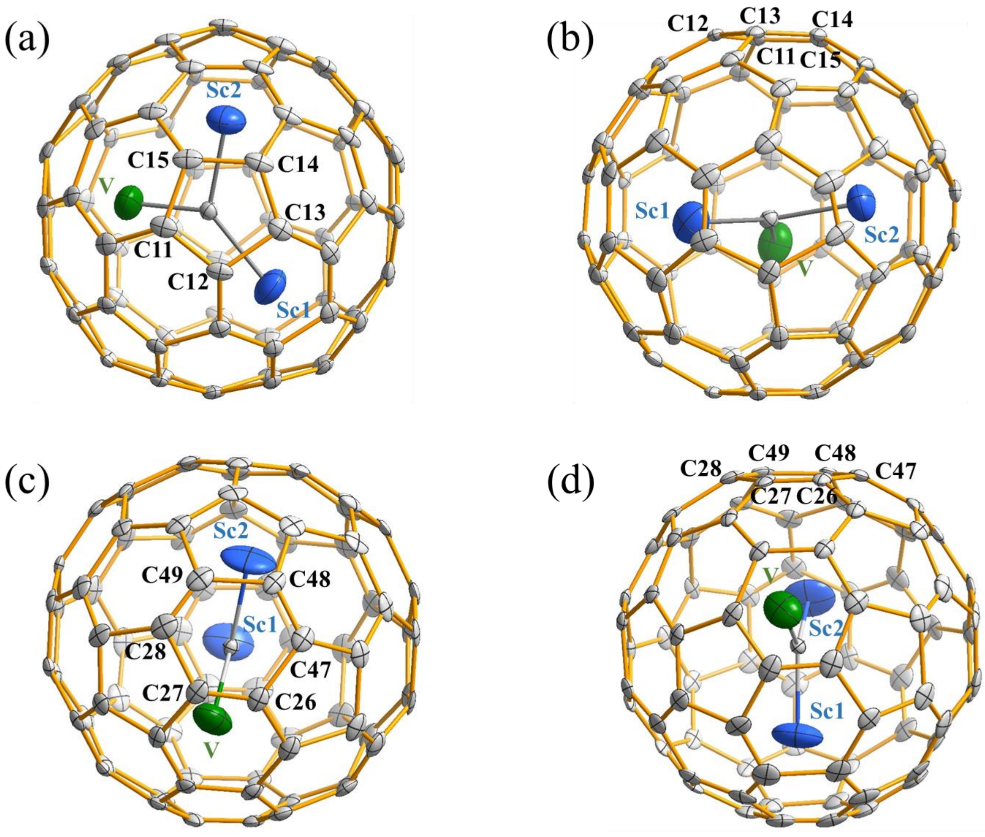

Supplementary Figure 8. (a, b) Two views of the $VSc_2C@D_{5h}(6)-C_{80}$. (a) Looking along the C_5 axis of cage. (b) Looking perpendicular to the C_5 axis of cage which is vertical in this view. (c, d) Two views of the $VSc_2C@D_{3h}(5)-C_{78}$. (c) Looking along the C_3 axis of cage. (d) Looking perpendicular to the C_3 axis of cage which is vertical in this view. The carbon atoms with labels show the location of the C_5/C_3 axis within $D_{5h}(6)-C_{80}/D_{3h}(5)-C_{78}$ cage.

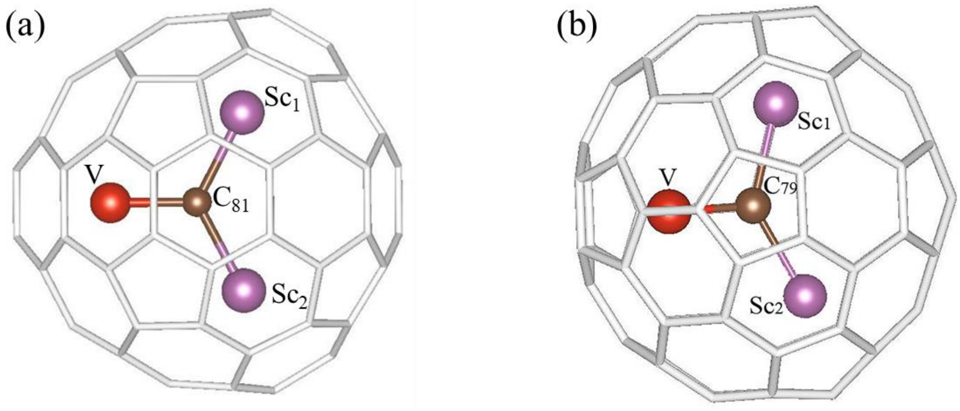

Supplementary Figure 9. The optimized geometrical configurations of (a) $VSc_2C@D_{5h}(6)\text{-}C_{80}$ and (b) $VSc_2C@D_{3h}(5)\text{-}C_{78}$.

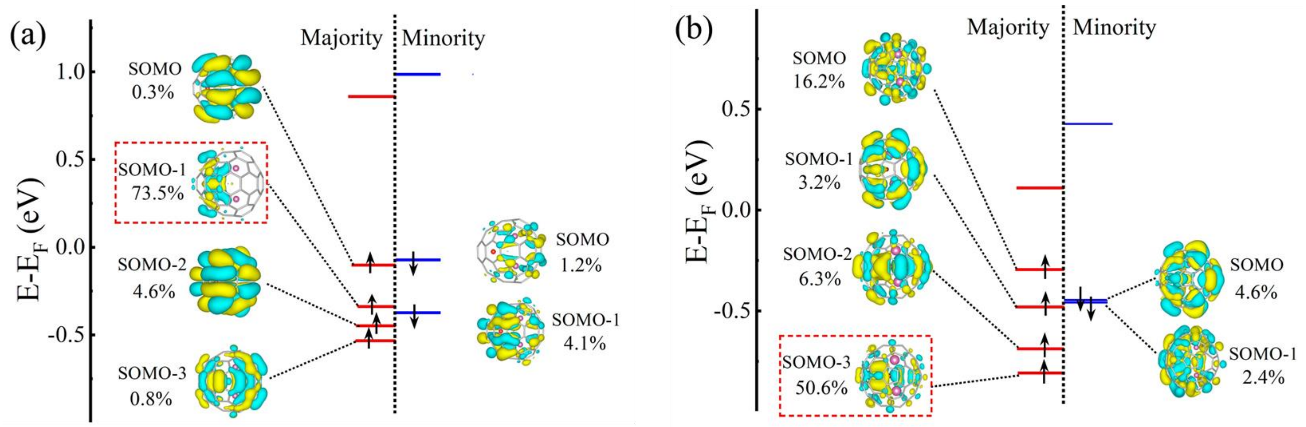

Supplementary Figure 10. The spin-resolved molecular levels and the spatial distribution of the frontier molecular orbitals of (a) $VSc_2C@D_{5h}(6)\text{-}C_{80}$ and (b) $VSc_2C@D_{3h}(5)\text{-}C_{78}$.

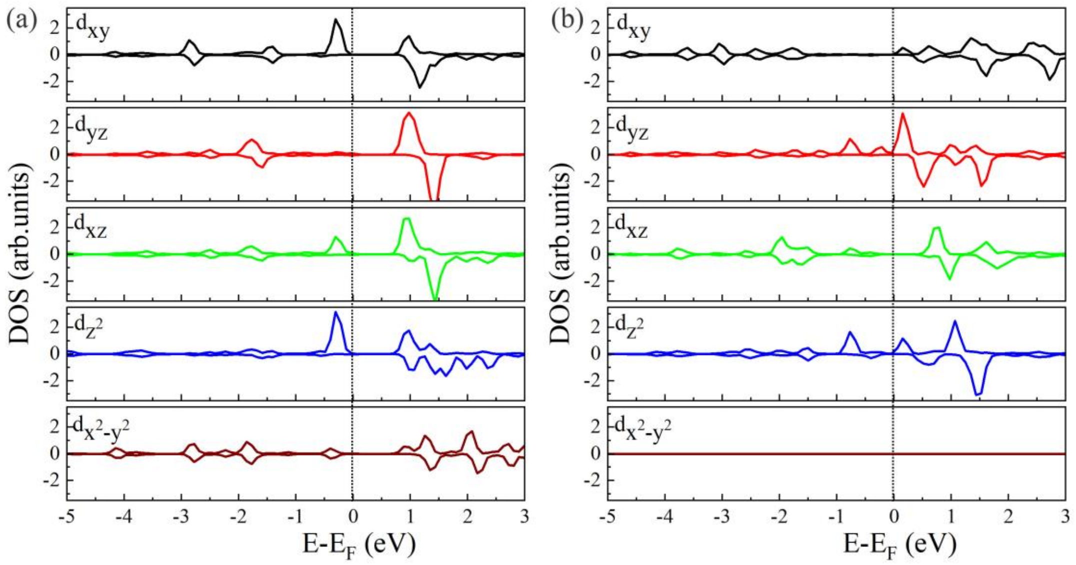

Supplementary Figure 11. The partial density of states (DOS) for V atoms in (a) $VSc_2C@D_{5h}(6)-C_{80}$ and (b) $VSc_2C@D_{3h}(5)-C_{78}$.

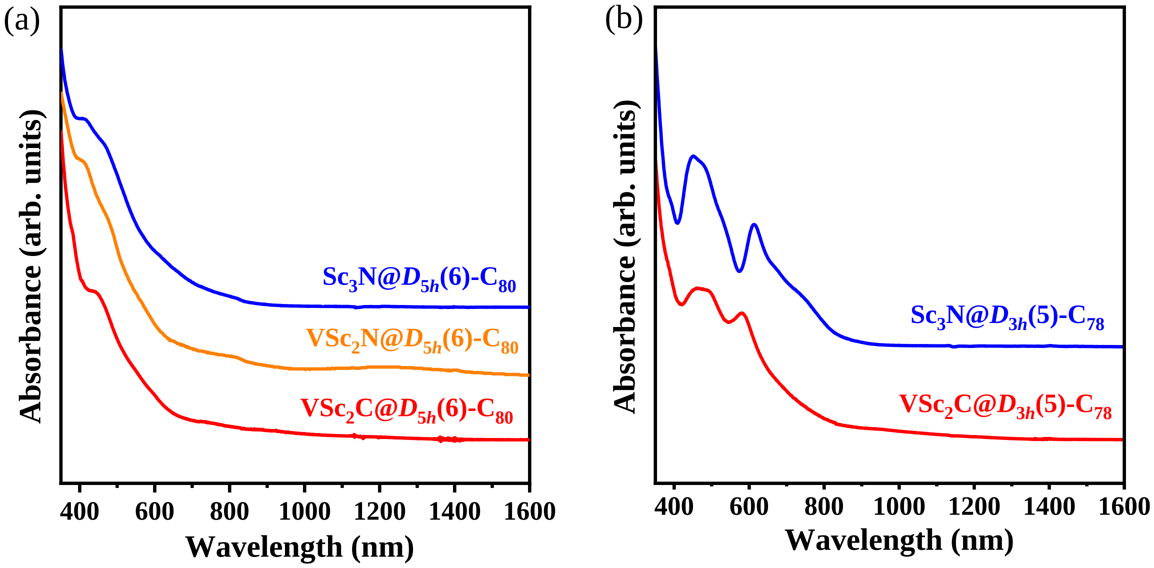

Supplementary Figure 12. UV-Vis–NIR spectra of (a) $VSc_2C@D_{5h}(6)-C_{80}$ dissolved in toluene in comparison with those of $VSc_2N@D_{5h}(6)-C_{80}$ and $Sc_3N@D_{5h}(6)-C_{80}$; (b) $VSc_2C@D_{3h}(5)-C_{78}$ dissolved in toluene in comparison with that of $Sc_3N@D_{3h}(5)-C_{78}$.

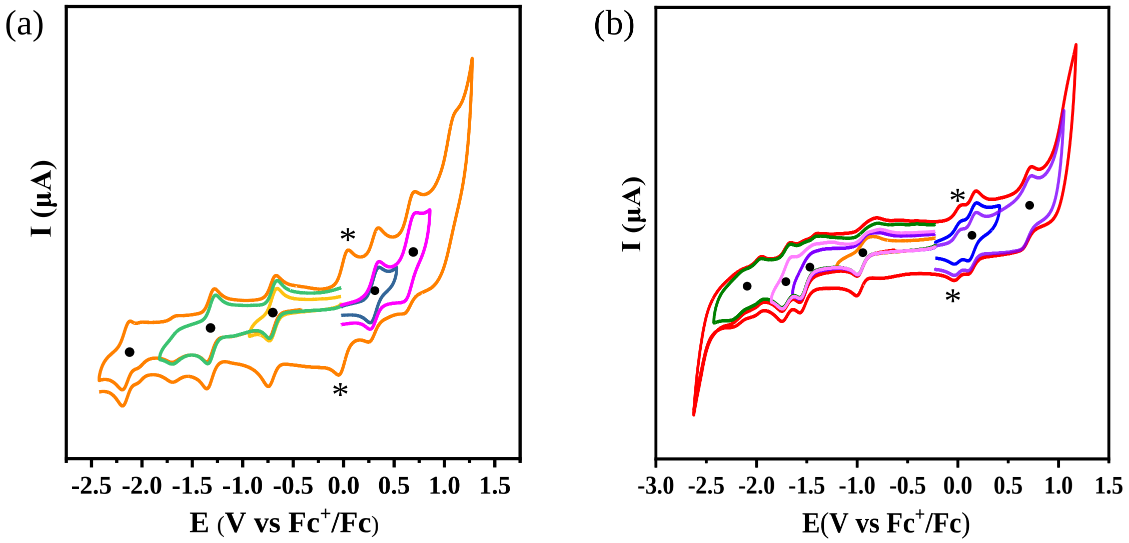

Supplementary Figure 13. Cyclic voltammograms of $VSc_2C@$*D_{5h}*$(6)-C_{80}$ (a) and $VSc_2C@$*D_{3h}*$(5)-C_{78}$ (b) in *o*-DCB solution in different scanning regions showing the correlation of each reduction/oxidation step. Scan rate: 100 mV/s, $TBAPF_6$ as supporting electrolyte. The half-wave potentials ($E_{1/2}$) of each redox step are marked with a solid dot to aid comparison. The asterisks label the oxidation and reduction peaks of ferrocene.

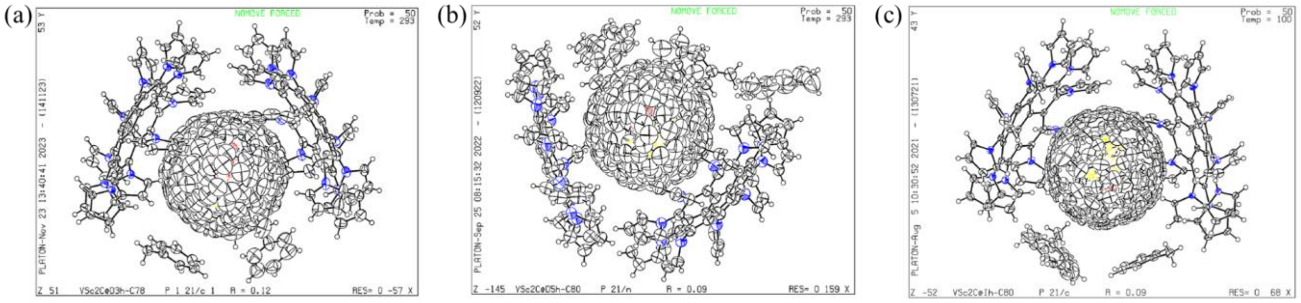

Supplementary Figure 14. ORTER-style illustration with probability ellipsoids for (a) $VSc_2C@D_{3h}(5)$-C_{78} (CCDC-2209496), (b) $VSc_2C@D_{5h}(6)$-C_{80} (CCDC-2038584), (c) $VSc_2C@I_h(7)$-C_{80} (CCDC-2038583).

Supplementary Table 1. Assignments of each subfraction.

Fraction	Subfraction	Major component	Relative abundance
**A**	**A-1**	C_{90}	66.3%
	**A-2**	Sc_2C_{84}	23.5%
	**A-3**	$Sc_3C_2@C_{80}$, $VSc_2C@I_h(7)$-C_{80}	10.2%
**A-3**	**A-3-1**	$VSc_2C@I_h(7)$-C_{80}	32.4%
	**A-3-2**	$Sc_3C_2@C_{80}$	67.6%
**B**	**B-1**	C_{94}	75.5%
	**B-2**	C_{96}	12.6%
	**B-3**	$VSc_2C@D_{5h}(6)$-C_{80}, $VSc_2C_2@I_h(7)$-C_{80}, $Sc_2C_{86\text{-}90}$	9.4%
	**B-4**	Sc_2C_{92}	2.5%
**B-3**	**B-3-1**	$VSc_2C@D_{5h}(6)$-C_{80}, $VSc_2C_2@I_h(7)$-C_{80}	7.7%
	**B-3-2**	Sc_2C_{86}	7.6%
	**B-3-3**	Sc_2C_{88}	28.4%
	**B-3-4**	Sc_2C_{90}	56.3%
**B-3-1**	**B-3-1-1**	$VSc_2C@D_{5h}(6)$-C_{80}	73.8%
	**B-3-1-2**	$VSc_2C_2@I_h(7)$-C_{80}	26.2%
**C**	**C-1**	C_{86}, $Sc_4C_2@C_{80}$	7.3%
	**C-2**	Sc_2C_{82}, $Sc_2O@C_{80}$	9.3%
	**C-3**	Sc_2C_{84}, $Sc_2O@C_{80}$, $VSc_2C@D_{3h}(5)$-C_{78}	59.3%
	**C-4**	$Sc_3N@C_{80}$, $Sc_2O@C_{82}$	24.1%
**C-3**	**C-3-1**	$VSc_2C@D_{3h}(5)$-C_{78}, $Sc_3N@D_{3h}(5)$-C_{78}	1.1%
	**C-3-2**	$Sc_2O@C_{80}$, $Sc@C_{82}$	7.0%
	**C-3-3**	Sc_2C_{84}, $Sc_2O@C_{82}$	91.9%
**C-3-1**	**C-3-1-1**	$Sc_3N@D_{3h}(5)$-C_{78}	14.1%
	**C-3-1-2**	$VSc_2C@D_{3h}(5)$-C_{78}	85.9%

The relative yields of $VSc_2C@D_{3h}(5)$-C_{78}, $VSc_2C@D_{5h}(6)$-C_{80} and $VSc_2C@I_h(7)$-C_{80} are estimated based on the integration areas of the corresponding peaks in the chromatograms shown in the Supplementary Fig. 2-4. Given that the relative yield of fraction **A** : **B** : **C** is 1.15 : 1 : 0.78, the relative yields of $VSc_2C@D_{3h}(5)$-C_{78}, $VSc_2C@D_{5h}(6)$-C_{80} and $VSc_2C@I_h(7)$-C_{80} can be calculated as:

$VSc_2C@D_{3h}(5)$-C_{78} : $VSc_2C@D_{5h}(6)$-C_{80} = (0.78 × 59.3% × 1.1% × 85.9%) : (1 × 9.4% × 7.7% × 73.8%) = 0.8 : 1.

$VSc_2C@I_h(7)$-C_{80} : $VSc_2C@D_{5h}(6)$-C_{80} = (1.15 × 10.2% × 32.4%) : (1 × 9.4% × 7.7% × 73.8%) = 7.1 : 1

Supplementary Table 2. Crystallographic data of $VSc_2C@D_{3h}(5)$-C_{78} and $VSc_2C@D_{5h}(6)$-C_{80} in comparison with that of $VSc_2C@I_h(7)$-C_{80}.

	$VSc_2C@D_{3h}(5)$-C_{78} ·2(DPC)·3(C_7H_8)	$VSc_2C@D_{5h}(6)$-C_{80} ·2(DPC)·4(C_7H_8)	$VSc_2C@I_h(7)$-C_{80} ·2(DPC)·3(C_7H_8)
Formula	C220 H104 N20 Sc2 V	C229 H112 N20 Sc2 V	C222 H104 N20 Sc2 V
Formula weight	3168.09	3284.25	3192.11
Crystal system	monoclinic	monoclinic	monoclinic
Space group	$P2_1/c$	$P2_1/n$	$P2_1/c$
a, Å	14.670	14.686	14.712
b, Å	31.810	32.049	32.026
c, Å	32.263	32.532	32.145
α, deg	90	90	90
β, deg	101.83	104.64	101.62
γ, deg	90	90	90
Volume, $Å^3$	14735.838	14928.090	14835.238
Z	4	4	4
ρ, g/cm^3	1.428	1.472	1.429
μ, mm^{-1}	0.225	0.227	0.225
R1 [reflections with $I>2\sigma(I)$]	0.1205	0.0865	0.0854
wR2 (all data)	0.3524	0.2634	0.2313

Supplementary Table 3. The fractional occupancies of the V/Sc positions within $VSc_2C@D_{5h}(6)-C_{80}$ and $VSc_2C@D_{3h}(5)-C_{78}$ in comparison with that of $VSc_2C@I_h(7)-C_{80}$.

Clusterfullerene	Fractional occupancy of metal positions			
	V1	V1a	V1b	V1c
	0.353(2)	0.308(2)	0.171(2)	0.168(2)
	Sc1	Sc1a	Sc1b	Sc1c
$VSc_2C@D_{5h}(6)-C_{80}$	0.353(2)	0.308(2)	0.171(2)	0.168(2)
	Sc2	Sc2a	Sc2b	Sc2c
	0.353(2)	0.308(2)	0.171(2)	0.168(2)
	V1	V1a	V1b	V1c
	0.314(3)	0.252(3)	0.233(3)	0.201(3)
	Sc1	Sc1a	Sc1b	Sc1c
$VSc_2C@D_{3h}(5)-C_{78}$	0.314(3)	0.252(3)	0.233(3)	0.201(3)
	Sc2	Sc2a	Sc2b	Sc2c
	0.314(3)	0.252(3)	0.233(3)	0.201(3)
	V1	V1a	V1b	
	0.458(2)	0.279(2)	0.263(2)	
$VSc_2C@I_h(7)-C_{80}$	Sc1	Sc1a	Sc1b	
	0.458(2)	0.279(2)	0.263(2)	
	Sc2	Sc2a	Sc2b	
	0.458(2)	0.279(2)	0.263(2)	

Supplementary Table 4. The V-C and Sc-C bond lengths (Å) based on different V and Sc sites within $VSc_2C@D_{5h}(6)-C_{80}$, $VSc_2C@D_{3h}(5)-C_{78}$ and $VSc_2C@I_h(7)-C_{80}$.

	$VSc_2C@D_{5h}(6)-C_{80}$		$VSc_2C@D_{3h}(5)-C_{78}$		$VSc_2C@I_h(7)-C_{80}$	
V-C	V1-C81	1.885(6)	V1-C79	1.867(8)	V1-C81	1.877(5)
	V1a-C81	1.923(5)	V1a-C79	1.909(7)	V1a-C81	1.874(5)
	V1b-C81	1.905(10)	V1b-C79	1.869(8)	V1b-C81	1.798(5)
	V1c-C81	1.812(11)	V1c-C79	1.884(9)		
Sc1-C	Sc1-C81	2.078(6)	Sc1-C79	2.054(10)	Sc1-C81	2.102(5)
	Sc1a-C81	2.226(6)	Sc1a-C79	2.121(9)	Sc1a-C81	2.100(6)
	Sc1b-C81	2.264(7)	Sc1b-C79	2.097(8)	Sc1b-C81	2.147(7)
	Sc1c-C81	2.162(9)	Sc1c-C79	2.059(12)		
Sc2-C	Sc2-C81	2.186(5)	Sc2-C79	2.055(9)	Sc2-C81	2.145(4)
	Sc2a-C81	1.956(6)	Sc2a-C79	1.991(7)	Sc2a-C81	2.096(6)
	Sc2b-C81	1.920(9)	Sc2b-C79	2.077(8)	Sc2b-C81	2.207(5)
	Sc2c-C81	2.176(8)	Sc2c-C79	2.022(10)		

Like $VSc_2C@I_h(7)-C_{80}$, the lengths of vanadium-carbon bonds in other minor cluster orientations within $VSc_2C@D_{5h}(6)-C_{80}$ and $VSc_2C@D_{3h}(5)-C_{78}$ also fall into the range of the V=C double bond lengths, further confirming the existence of V=C double bonds.

Supplementary Table 5. Comparison of three possible conformations of VSc_2C@D_{3h}(5)-C_{78}.

	VSc_2C@D_{3h}(5)-C_{78}-**A**	VSc_2C@D_{3h}(5)-C_{78}-**B**	VSc_2C@D_{3h}(5)-C_{78}-**C**
Molecular structure	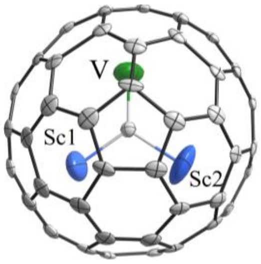	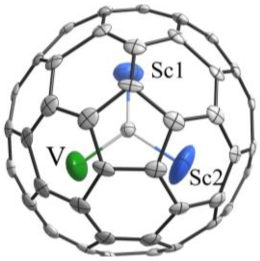	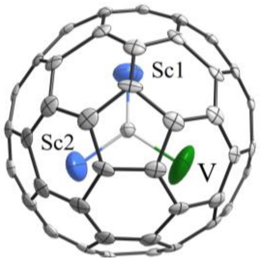
Relative energy (eV)	0	0.09	0.08
R1 (%)	12.05	12.17	12.09
wR2 (%)	35.24	35.51	35.35

Since Sc and V atoms are close in the d-block transition metal series with comparable atomic sizes and scattering powers, it is difficult to determine the exact positions of the encapsulated V and Sc atoms merely based on the crystallographic data. Alternatively, we managed to distinguish V and Sc atoms based on a comparison of the the R1/wR2 values obtained from different conformations of the encapsulated VSc_2C cluster (**A**, **B**, **C**) combined with DFT calculations. There are three possible conformations of the encapsulated VSc_2C cluster within VSc_2C@D_{3h}(5)-C_{78} (**A**, **B**, **C**). Based on the DFT calculation results, VSc_2C@D_{3h}(5)-C_{78}-**A** in which V possesses the shorter bond lengths to the central carbon atom (around 1.8 Å) has the lowest relative energy with the smallest R1 and wR2 values (12.05%/35.24%). Besides, R1 and wR2 values both increase when changing the disordered V sites to other sites with longer bond length of approximately 2 Å with the central carbon (VSc_2C@D_{3h}(5)-C_{78}-**B** and VSc_2C@D_{3h}(5)-C_{78}-**C**, Supplementary Table 3). Hence, it is concluded that VSc_2C@D_{3h}(5)-C_{78}-**A** is the most stable conformation, in which the short V-C bond is attributed to V=C double bond and the two Sc-C bonds are in the form of single bonds.

Supplementary Table 6. Comparison of three possible conformations of VSc_2C@D_{5h}(6)-C_{80}.

	VSc_2C@D_{5h}(6)-C_{80}-**A**	VSc_2C@D_{5h}(6)-C_{80}-**B**	VSc_2C@D_{5h}(6)-C_{80}-**C**
Molecular structure	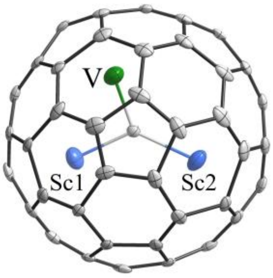	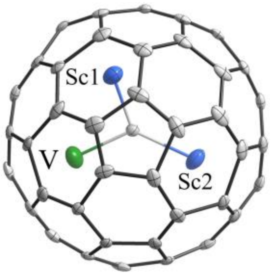	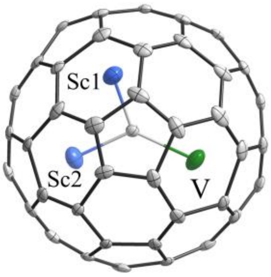
Relative energy (eV)	0	0.22	0.21
R1 (%)	8.65	8.87	8.82
wR2 (%)	26.34	27.65	27.85

Similar to the case of VSc_2C@D_{3h}(5)-C_{78}, VSc_2C@D_{5h}(6)-C_{80}-**A** bearing the V=C double is the most stable conformation with the lowest relative energy and the smallest R1 and wR2 values (8.65%/26.34%).

Supplementary Table 7. The lengths (Å) of metal-carbon bonds within the major VSc_2C cluster in VSc_2C@D_{3h}(5)-C_{78}, VSc_2C@D_{5h}(6)-C_{80} and VSc_2C@I_h(7)-C_{80}, compared with the lengths of metal-nitrogen bonds within the corresponding M_3N@C_{80} and M_3N@C_{78} counterparts.

	VSc_2C@D_{3h}(5)-C_{78}	VSc_2C@D_{5h}(6)-C_{80}	VSc_2C@I_h(7)-C_{80}
V-C	1.867(8)	1.885(6)	1.877(5)
Sc1-C	2.054(10)	2.078(6)	2.102(5)
Sc2-C	2.055(9)	2.186(5)	2.145(4)
	VSc_2N@I_h(7)-C_{80} (ref 2)	VSc_2N@D_{5h}(6)-C_{80} (ref 3)	V_2ScN@I_h(7)-C_{80} (ref 2)
V1-N	1.858(7)	1.900(5)	1.995(5)
V2-N	–	–	2.027(3)
Sc1-N	2.036(4)	2.071(2)	2.003(6)
Sc2-N	2.036(4)	2.071(2)	–
	Sc_3N@D_{3h}(5)-C_{78} (ref 4)	Sc_3N@D_{5h}(6)-C_{80} (ref 5)	Sc_3N@I_h(7)-C_{80} (ref 6)
Sc1-N	1.9998(10)	2.014(2)	1.9931(14)
Sc2-N	2.0106(10)	2.031(2)	2.0323(16)
Sc3-N	2.0111(10)	2.041(2)	2.0526(14)

Supplementary Table 8. The relative total energy (ΔE, eV) of $MSc_2C@C_{2n}$ (M = V, Ti, U, 2n = 78, 80) with different electronic configurations and M=C bonds of MSc_2C cluster.

	$MSc_2C@I_h(7)$-C_{80}-**A** [8e; B.N.=4; V.S.=-4][a]	$MSc_2C@I_h(7)$-C_{80}-**B** [8e; B.N.=3; V.S.=-4][a]	$MSc_2C@I_h(7)$-C_{80}-**C** [7e; B.N.=4; V.S.=-3][a]	$MSc_2C@I_h(7)$-C_{80}-**D** [7e; B.N.=3; V.S.=-3][a]
Electronic configuration	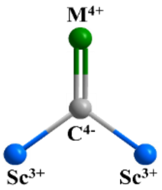	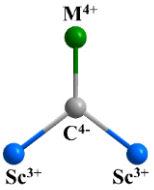	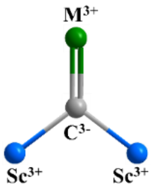	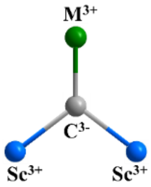
$VSc_2C@D_{3h}(5)$-C_{78}	0	0.14	0.69	0.93
$VSc_2C@D_{5h}(6)$-C_{80}	0	0.37	0.78	1.23
$VSc_2C@I_h(7)$-C_{80}	0	0.38	0.47	1.09
$TiSc_2C@I_h(7)$-C_{80}	0	0.45	0.63	1.12
$USc_2C@I_h(7)$-C_{80}	0	0.10	0.61	0.69

[a] 8e/7e means the total of valence electron of C^{4-}/C^{3-} anion; B.N. and V.S. represent bond number and valence state, respectively. Except for the 8e-rule, the structure is stable when B.N. + V.S.=0 (electroneutral). Hence, configurations **B, C, D** are all less stable than configuration **A**.

Supplementary Table 9. The relative total energy (ΔE, eV) of isolated $TiM_2C@C_{2n}$ (M = Lu, Y, Nd, Gd, Tb, Dy, Er, Sc, 2n = 78, 80) with different electronic configurations and M=C bonds of TiM_2C cluster.

	$TiM_2C@C_{2n}$-**A** [8e; B.N.=4; V.S.=-4][a]	$TiM_2C@C_{2n}$-**B** [8e; B.N.=3; V.S.=-4][a]	$TiM_2C@C_{2n}$-**C** [7e; B.N.=4; V.S.=-3][a]	$TiM_2C@C_{2n}$-**D** [7e; B.N.=3; V.S.=-3][a]
Electronic configuration	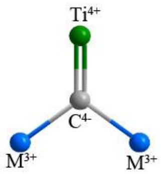	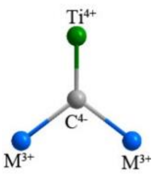	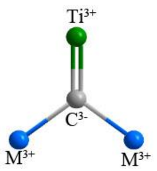	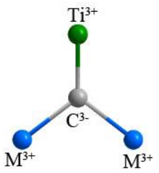
$TiLu_2C@I_h(7)$-C_{80}	0	1.94	0.09	2.37
$TiY_2C@I_h(7)$-C_{80}	0	0.39	0.48	0.83
$TiNd_2C@I_h(7)$-C_{80}	0	1.85	0.49	2.24
$TiGd_2C@I_h(7)$-C_{80}	0	2.28	0.63	2.71
$TiTb_2C@I_h(7)$-C_{80}	0	2.00	0.25	2.13
$TiDy_2C@I_h(7)$-C_{80}	0	2.37	0.54	2.56
$TiEr_2C@I_h(7)$-C_{80}	0	2.13	0.55	2.43
$TiDyYC@I_h(7)$-C_{80}	0	2.09	0.43	2.29
$TiDy_2C@D_{5h}(6)$-C_{80}	0	2.07	0.42	2.39
$TiSc_2C@D_{5h}(6)$-C_{80}	0	0.95	0.39	2.36
$TiSc_2C@D_{3h}(5)$-C_{78}	0	1.26	0.36	2.49

[a] 8e/7e means the total of valence electron of C^{4-}/C^{3-} anion; B.N. and V.S. represent bond number and valence state, respectively. Except for the 8e-rule, the structure is stable when B.N. + V.S.=0 (electroneutral). Hence, configurations **B, C, D** are all less stable than configuration **A**.

Supplementary Table 10. The relative energy (ΔE, eV) of $VSc_2N@I_h(7)$-C_{80} with different electronic configurations and V-N bonds of VSc_2N cluster.

ΔE (eV)	$VSc_2N@I_h(7)$-C_{80}-**A**<br>[9e; B.N.=4; V.S.=-4][a]	$VSc_2N@I_h(7)$-C_{80}-**B**<br>[9e; B.N.=3; V.S.=-4][a]	$VSc_2N@I_h(7)$-C_{80}-**C**<br>[8e; B.N.=4; V.S.=-3][a]	$VSc_2N@I_h(7)$-C_{80}-**D**<br>[8e; B.N.=3; V.S.=-3][a]
Electronic configuration	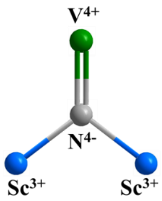	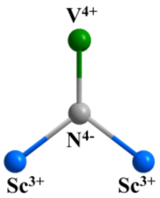	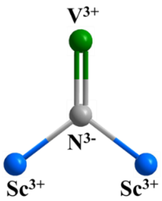	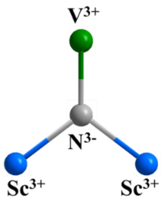
$VSc_2N@I_h(7)$-C_{80}	0.75	0.34	0.63	0

[a] 9e/8e means the total of valence electron of N^{4-}/N^{3-} anion; B.N. and V.S. represent bond number and valence state, respectively.

Supplementary Table 11. Electronic absorption data of $VSc_2C@$*D*$_{5h}$(6)-C_{80} and $VSc_2C@$*D*$_{3h}$(5)-C_{78} in comparison with those of $VSc_2N@$*D*$_{5h}$(6)-C_{80}, $Sc_3N@$*D*$_{5h}$(6)-C_{80}, $DySc_2N@$*D*$_{3h}$(5)-C_{78} and $Sc_3N@$*D*$_{3h}$(5)-C_{78}.

Sample	Vis-NIR absorption peaks (nm)	Absorption onset (λ_{onset}, nm)	$\Delta E_{gap,\ optical}$ / eV[a]	Ref
$VSc_2C@D_{5h}(6)\text{-}C_{80}$	383, 447	1490	0.83	This work
$VSc_2N@D_{5h}(6)\text{-}C_{80}$	413, 475	1900	0.65	3
$Sc_3N@D_{5h}(6)\text{-}C_{80}$	413, 472	950	1.30	7
$VSc_2C@D_{3h}(5)\text{-}C_{78}$	462, 581	1430	0.87	This work
$DySc_2N@D_{3h}(5)\text{-}C_{78}$	452, 545, 612, 726	1120	1.10	8
$Sc_3N@D_{3h}(5)\text{-}C_{78}$	460, 623	1040	1.19	9

[a] $\Delta E_{gap,\ optical}$ (optical bandgap) = 1240 / λ_{onset}.

Supplementary Table 12. Redox potentials (V vs. Fc+/Fc) and electrochemical gaps ($\Delta E_{gap,\ ec}$) of $VSc_2C@D_{5h}(6)-C_{80}$ and $VSc_2C@D_{3h}(5)-C_{78}$ in comparison with those of other analogous endohedral nitride clusterfullerenes.

	$^{red}E_1$	$^{red}E_2$	$^{red}E_3$	$^{red}E_4$	$^{ox}E_1$	$^{ox}E_2$	$\Delta E_{gap,EC}$ / V[a]	Ref
$VSc_2C@D_{5h}(6)-C_{80}$	-0.70	-1.31	-2.16	-	0.30	0.66	1.00	This work
$VSc_2N@D_{5h}(6)-C_{80}$	-0.78	-1.59	-2.01	-2.37	0.42	0.93	1.20	3
$Sc_3N@\ D_{5h}(6)-C_{80}$	-1.33[b]	-1.82	-	-	0.34	-	1.67	10
$VSc_2C@D_{3h}(5)-C_{78}$	-0.91	-1.49[b]	-1.71	-2.10[b]	0.14	0.69	1.05	This work
$Sc_3N@D_{3h}(5)-C_{78}$	-1.56[b]	-1.91[b]	-	-	0.21	0.68	1.77	11

[a] $\Delta E_{gap,\ EC}$ (electrochemical band-gap) = $^{ox}E_1$ − $^{red}E_1$, [b] Irreversible peak value.

Supplementary Table 13. Note and corresponding justification on CheckCif file B-level alerts of $VSc_2C@D_{3h}(5)-C_{78}$, $VSc_2C@D_{5h}(6)-C_{80}$ and $VSc_2C@I_h(7)-C_{80}$.

	Alert level B	Author Response
$VSc_2C@D_{3h}(5)-C_{78}$	Large U3/U1 Ratio for Average U(i,j) Tensor .... 4.4 Note	This is caused by the large amount of disorders in the structure.
$VSc_2C@D_{5h}(6)-C_{80}$	Poor Data / Parameter Ratio .... 7.94 Note	This is caused by the limited phi scan with synchrotron radiation X-ray diffraction facility.
$VSc_2C@I_h(7)-C_{80}$	–	–

References

1. Guan, R. et al. Self-driven carbon atom implantation into fullerene embedding metal-carbon cluster. *Proc. Natl. Acad. Sci. U. S. A*. **119,** e2202563119 (2022).
2. Wei, T. et al. Entrapping a Group-VB Transition Metal, Vanadium, within an Endohedral Metallofullerene: $V_xSc_{3-x}N$@I_h-C_{80} (x = 1, 2). *J. Am. Chem. Soc.* **138**, 207-214 (2016).
3. Wei, T. et al. Blending Non-Group-3 Transition Metal and Rare-Earth Metal into a C_{80} Fullerene Cage with D_{5h} Symmetry. *Angew. Chem. Int. Ed.* **57**, 10273-10277 (2018).
4. B. Q. Mercado *et al.*, A single crystal X-ray diffraction study of a fully ordered cocrystal of pristine Sc_3N@D_{3h}(5)–C_{78}. *Polyhedron* **58**, 129-133 (2013).
5. T. Cai *et al.*, Structure and Enhanced Reactivity Rates of the D_{5h} Sc_3N@C_{80} and Lu_3N@C_{80} Metallofullerene Isomers: The Importance of the Pyracylene Motif. *J. Am. Chem. Soc.* **128**, 8581-8589 (2006).
6. S. Stevenson *et al.*, Preparation and Crystallographic Characterization of a New Endohedral, Lu_3N@C_{80} · 5 (o-xylene), and Comparison with Sc_3N@C_{80} · 5 (o-xylene). *Chem. Eur. J.* **8**, 4528-4535 (2002).
7. Yang, S., Popov, A., Kalbac, M. & Dunsch, L. The isomers of gadolinium scandium nitride clusterfullerenes $Gd_xSc_{3-x}N$@C_{80} (x=1, 2) and their influence on cluster structure. *Chem. Eur. J.* **14**, 2084-2092 (2008).
8. Wei, T., Liu, F., Wang, S., Zhu, X., Popov, A. & Yang, S. An expanded family of dysprosium-scandium mixed-metal nitride clusterfullerenes: the role of the lanthanide metal on the carbon cage size distribution. *Chem. Eur. J.* **21**, 5750-5759 (2015).
9. Olmstead, M. M. et al. Isolation and structural characterization of the endohedral fullerene Sc_3N@C_{78}. *Angew. Chem. Int. Ed.* **40**, 1223 (2001).
10. Cai T, et al. Structure and Enhanced Reactivity Rates of the D_{5h} Sc_3N@C_{80} and Lu_3N@C_{80} Metallofullerene Isomers: The Importance of the Pyracylene Motif. *J. Am. Chem. Soc*. **128**, 8581-8589 (2006).
11. Beavers, C. M., Chaur, M. N., Olmstead, M. M., Echegoyen, L. & Balch, A. Large Metal Ions in a Relatively Small Fullerene Cage: The Structure of Gd_3N@C_2(22010)-C_{78} Departs from the Isolated Pentagon Rule. *J. Am. Chem. Soc.* **131**, 11519-11524 (2009).
